# Supplementary material for: TRIM28-Regulated Transposon Repression Is Required for Human Germline Competency and Not Primed or Naive Human Pluripotency
Source: Stem Cell Reports. 2017 Dec 28;10(1):243–56. doi: 10.1016/j.stemcr.2017.11.020 (PMC5768987; doi:10.1016/j.stemcr.2017.11.020)
Supplement: Document S1. Supplemental Experimental Procedures and Figures S1–S5 [file mmc1.pdf]

Stem Cell Reports, Volume *10*

Supplemental Information

TRIM28-Regulated Transposon Repression Is Required for Human Germline Competency and Not Primed or Naive Human Pluripotency

Yu Tao, Ming-Ren Yen, Tsotne Chitiashvili, Haruko Nakano, Rachel Kim, Linzi Hosohama, Yao Chang Tan, Atsushi Nakano, Pao-Yang Chen, and Amander T. Clark

Supplemental Figures

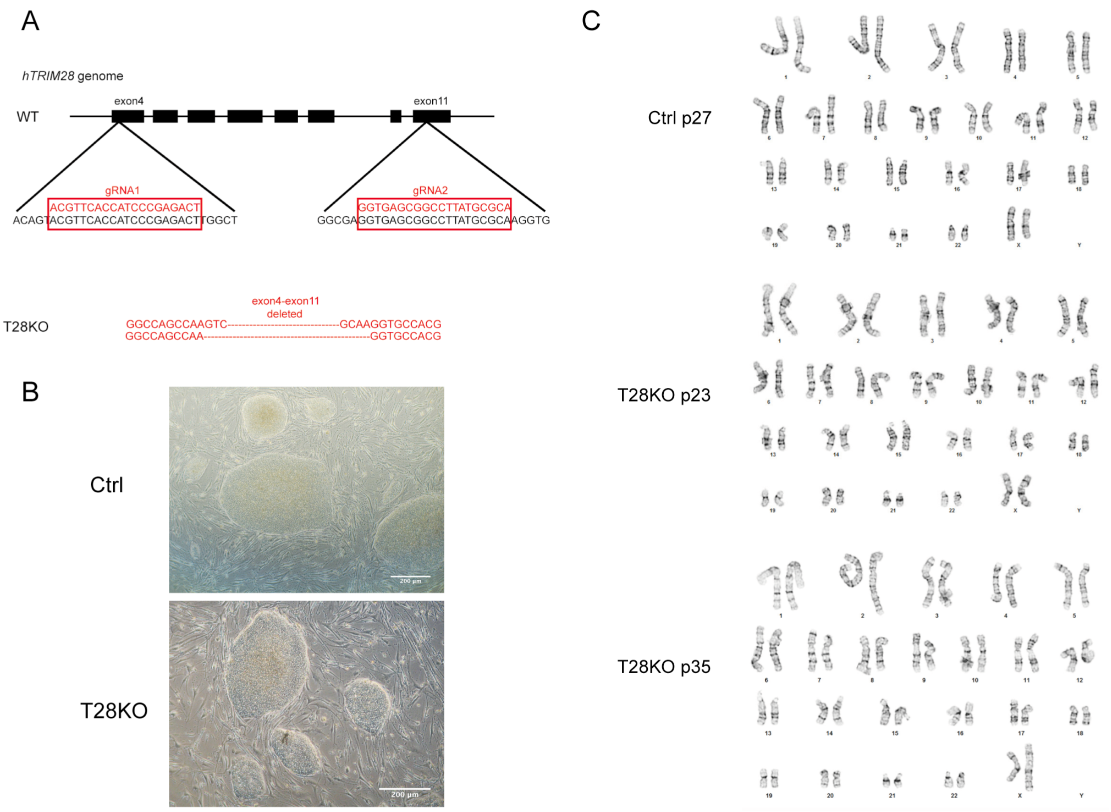

Figure S1 related to Figure 1
(A) Schematic overview of the TRIM28 locus and the CRISPR/Cas9 gRNA targeting sites. Guide RNA-targeting sequences are marked as red in the box. The TRIM28 mutation was confirmed by Sanger Sequencing DNA clones from the T28KO (U1-9) mutant cell line.
(B) Bright field images of primed Ctrl hESC and T28KO (U1-9) hESC, scale bar indicates 100µm.
(C) Karyotypes of Ctrl (passage number: 27) and T28KO (U1-9) (passage number: 23 and 35) primed hESCs.

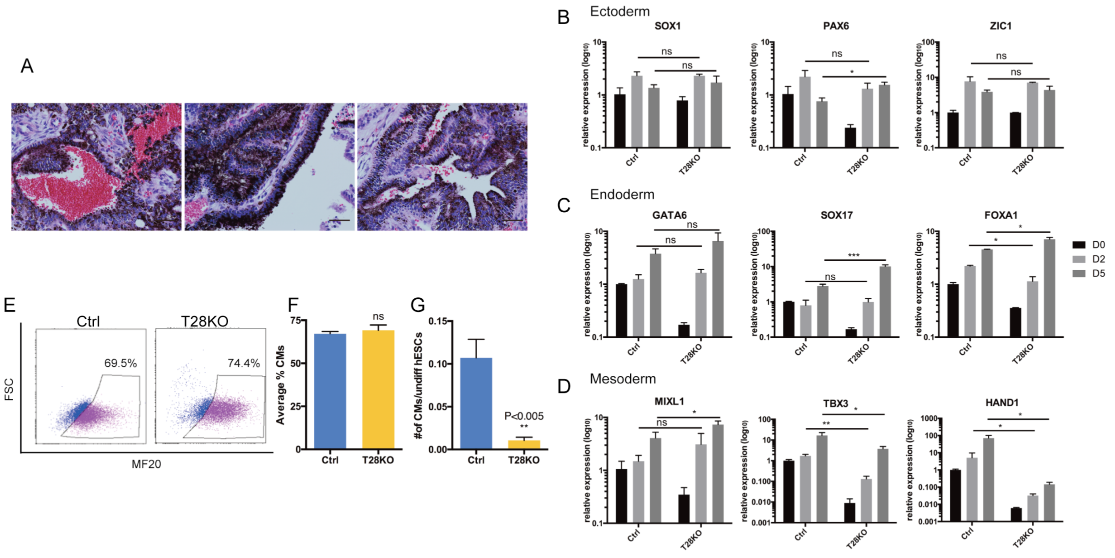

Figure S2 related to Figure 2

(A) Representative photos of teratomas from primed T28KO (U1-9) hESCs, detailed (hematoxylin and eosin) HE staining images show the highly pigmented cells in T28KO (U1-9) teratoma, scale bar = 50μm.

(B-D) RT-qPCR of representative genes of the 3 somatic germ layers, in undifferentiated hESC (D0), Day2, and Day5 of spontaneous EB differentiation from both Ctrl and T28KO (U1-9) hESCs, n=3 independent experiments, * indicates $p<0.05$, ** indicates $p<0.01$, ns= non significant, error bar = SD.

(E-G) Cardiomyocyte (CM) differentiation of Ctrl and T28KO (U1-9) hESC. (E) Representative flow cytometry results of Day14 EB, MF20 is a positive marker of CMs at this stage. (F) Average percentage of CM at Day14, n=3 independent experiments, $p>0.05$ by Student's t-test. (G) CM differentiation efficiency when the cell numbers of acquired CMs at Day 14 are normalized to the cell numbers of starting undifferentiated hESCs, error bar = SD.

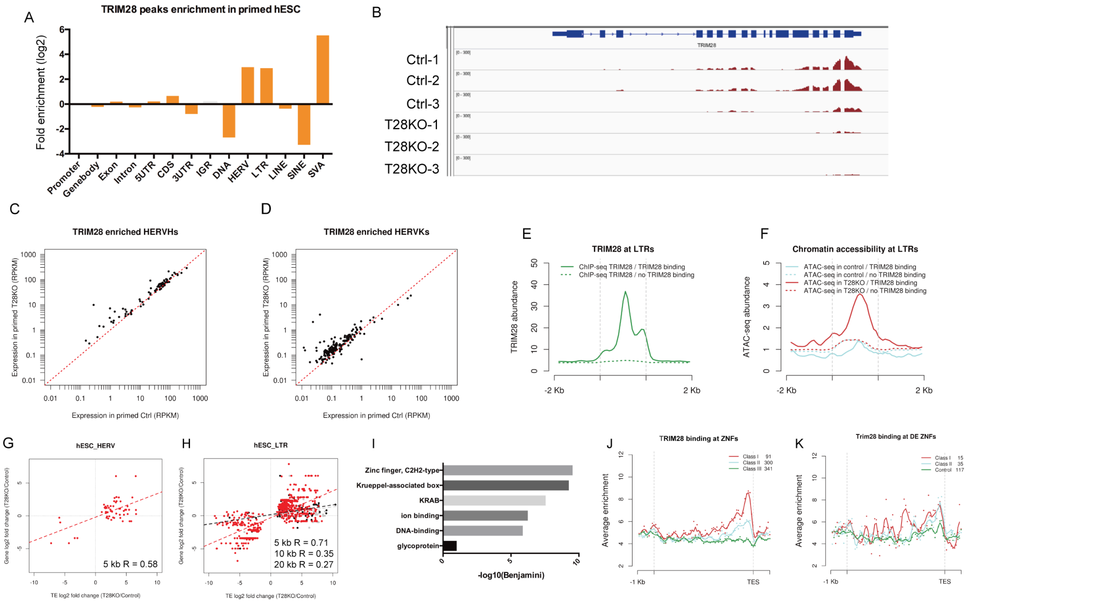

Figure S3 related to Figure 3

(A) Enrichment analysis of TRIM28 binding sites in wild type primed hESCs of ChIP-Seq data set from Theunissen et al.).

(B) Snapshots of RNA-seq peaks of Ctrl and T28KO (U1-9) primed hESCs at the TRIM28 locus, each with 3 replicates.

(C-D) Scatter plots of relative expression shown in Reads Per Kilobase of transcript per Million mapped reads (RPKM) of HERVH and HERVK in Ctrl and T28KO (U1-9) primed hESCs.

(E-F) Meta-plot of TRIM28 binding at LTRs (Meta analysis from the data set of Theunissen et al.) and chromatin accessibility at TRIM28 bound LTRs in Ctrl and T28KO (U1-9) hESCs.

(G-H) Correlation between differentially expressed HERVs / LTRs and differentially expressed neighboring genes.

(I) Gene ontology analysis of up-regulated genes in T28KO (U1-9) primed hESCs.

(J) TRIM28 binding at ZNFs in primed hESCs using the class I, class II and class III defined ZNFs identified by Valle-Garcia et al., in the human erythroleukemic cell line K562.

(K) Up-regulated ZNFs identified in T28KO (U1-9) hESCs correspond to class I and class II ZNFs, with Trim28 binding in the middle and 3’ end of the gene.

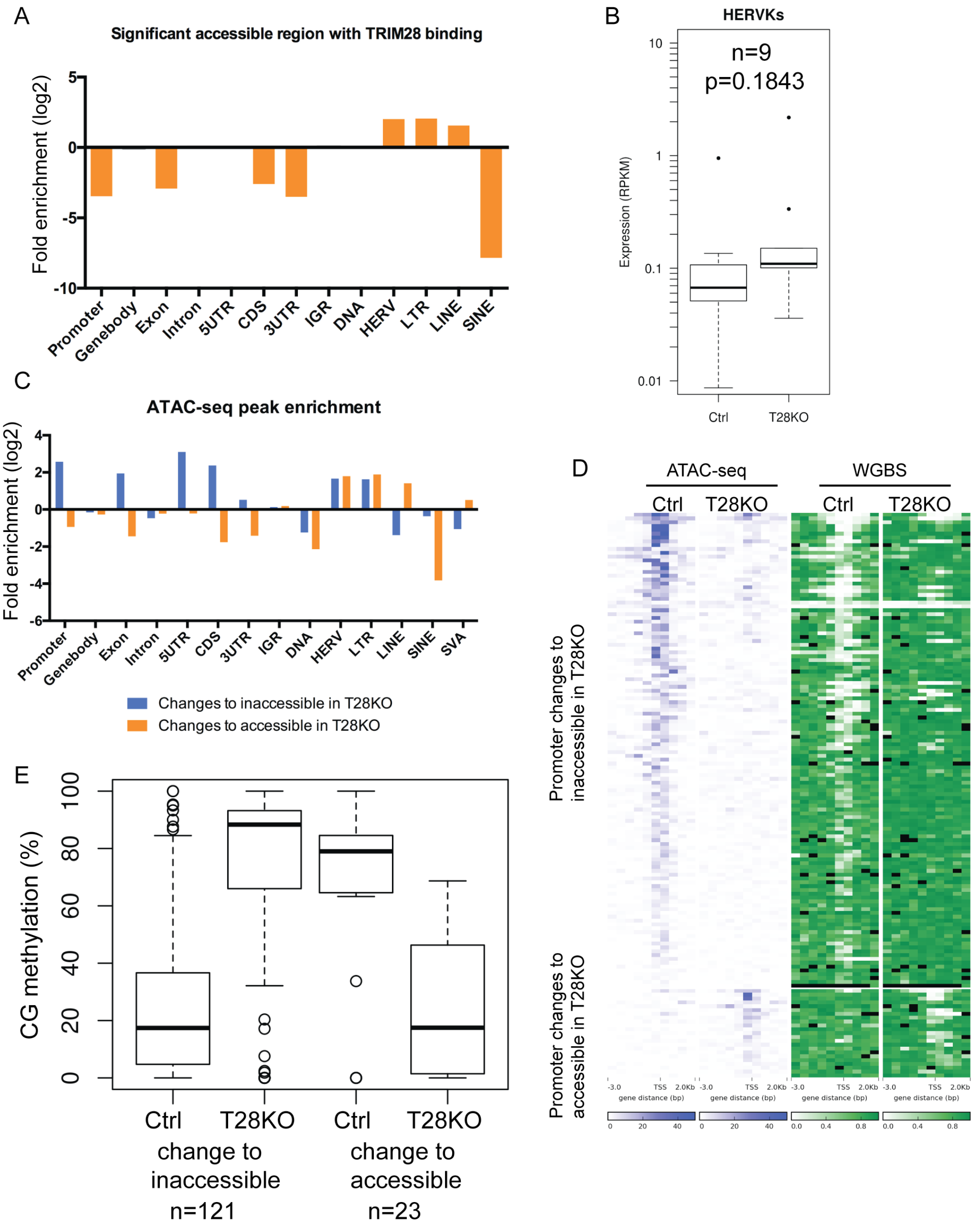

Figure S4 related to Figure 4

(A) Enrichment analysis of the regions bound by TRIM28 in wild type hESCs that become accessible in T28KO (U1-9) hESCs relative to Ctrl.

(B) Box-plot of the expression levels of the expressed HERVKs from Figure 4D.

(C) Enrichment analysis of the regions that changed from accessible to inaccessible in T28KO (U1-9) hESCs relative to Ctrl and from inaccessible to accessible in T28KO (U1-9) hESCs relative to Ctrl.

(D) Heatmap of promoters that change from accessible to inaccessible (n=121) and inaccessible to accessible (n=23) by ATAC-Seq in T28KO (U1-9) hESCs relative to Ctrls and the corresponding DNA methylation levels.

(E) Box-plot of percent CG DNA methylation of the same promoters from Figure 4D.

A

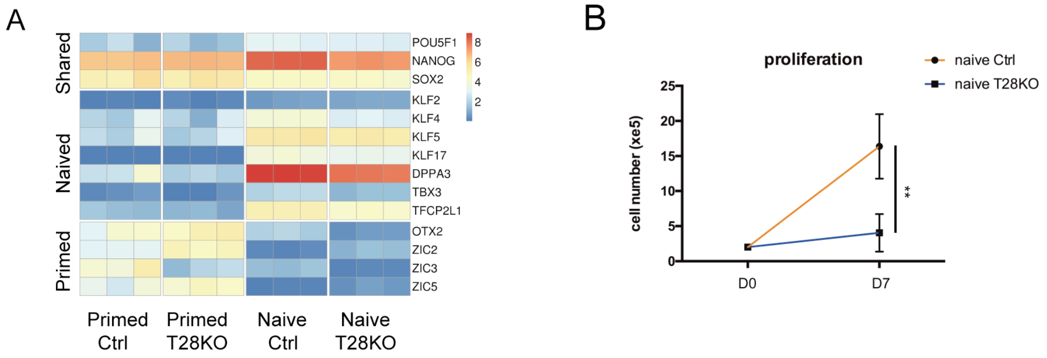

C

Ctrl

Amp/Deletion Table									
Chr	Amp/Del	Start (bp)	Stop (bp)	Size(kb)	Chr Band	# Probes	Log2 Ratio	Genes$^{\alpha}$	Overlap Normal CNVs?$^{\beta}$
6	Amp	168,371,416	168,581,212	210	q27	7	0.677991	MLLT4, HGC6.3, KIF25-AS1, KIF25, FRMD1	Yes
15	Amp	20,481,702	21,939,711	1,458	q11.1 - q11.2	5	0.522967	CHEK2P2, HERC2P3, GOLGA6L6, GOLGA8CP, NBEAP1, MIR3118-4, MIR3118-3, MIR3118-2, POTEB3, POTEB, POTEB2, NF1P2, MIR5701-3, MIR5701-1, MIR5701-2, LINC01193, LOC646214	Yes

Total Amp/Del: 2

T28KO

Amp/Deletion Table									
Chr	Amp/Del	Start (bp)	Stop (bp)	Size(kb)	Chr Band	# Probes	Log2 Ratio	Genes$^{\alpha}$	Overlap Normal CNVs?$^{\beta}$
6	Amp	168,371,416	168,581,212	210	q27	7	0.507282	MLLT4, HGC6.3, KIF25-AS1, KIF25, FRMD1	Yes
15	Amp	20,481,702	21,939,711	1,458	q11.1 - q11.2	5	0.453266	CHEK2P2, HERC2P3, GOLGA6L6, GOLGA8CP, NBEAP1, MIR3118-4, MIR3118-3, MIR3118-2, POTEB3, POTEB, POTEB2, NF1P2, MIR5701-3, MIR5701-1, MIR5701-2, LINC01193, LOC646214	Yes
20	Del	40,703,506	41,704,257	1,001	q12 - q13.11	20	-0.30984	PTPRT, LOC101927159	No
22	Del	47,716,755	49,920,184	2,203	q13.31 - q13.33	36	-0.27632	LL22NC03-75H12.2, LINC00898, LOC284930, MIR3201, FAM19A5, LOC284933, MIR4535, LINC01310, C22orf34	No

Total Amp/Del: 4

D

	All expressed	Primed TRIM28 enriched	Naive TRIM28 enriched
HERVH	1400	81	891
HERVK	622	169	287
SVA	2496	293	1225
LINE	139634	1055	5107

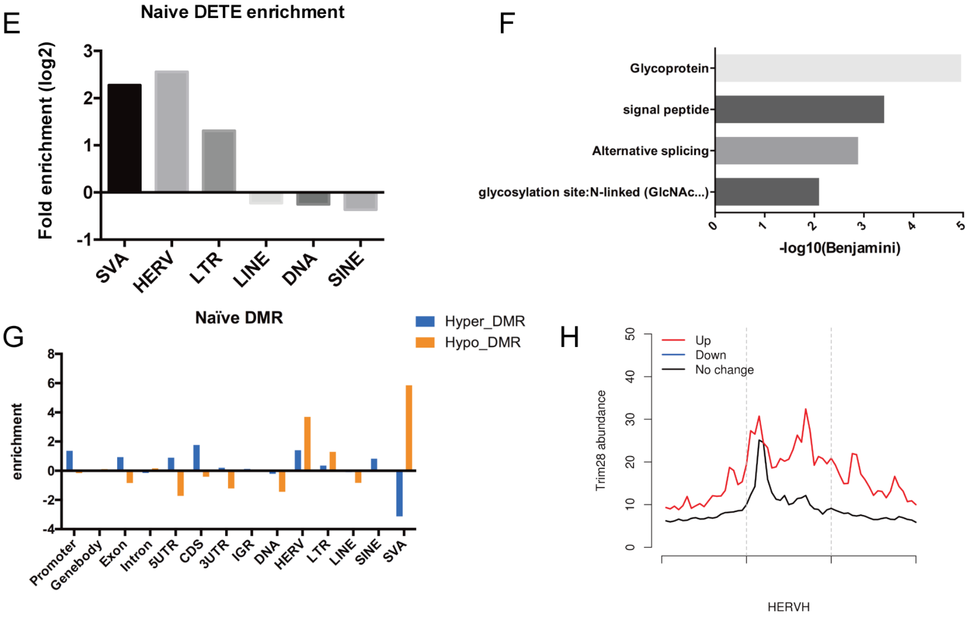

Figure S5 related to Figure 6

(A) Heatmap of diagnostic genes that were previously reported (Pastor et al., 2016) as being up-regulated in either the primed or naïve state as well as genes that are shared by both states.

(B) Average cell counts of over seven days of Ctrl (Ctrl UCLA1) and T28KO (T28KO(U1-9)) naïve hESCs (n=3 independent experiments), ** indicates $p<0.01$, error bar = SD.

(C) Copy Number Variants (CNV) analysis of naïve Ctrl and T28KO (U1-9) hESCs, naïve Ctrl hESCs have 2 CNVs which are overlapped with normal CNVs, naïve T28KO (U1-9) hESCs have another 2 deletions that are not overlapped with normal CNVs.

(D) Table shows numbers of all expressed HERVH, HERVK, SVA and LINE that are also bound by TRIM28 in control primed and control naïve hESCs using ChIP-Seq as defined by (Theunissen et al., 2016).

(E) Enrichment analysis of the DETE between Ctrl and T28KO (U1-9) naive hESC. Fold enrichment is represented as log2 (T28KO/Ctrl).

(F) Gene ontology analysis of up-regulated genes in T28KO (U1-9) naïve hESCs relative to Ctrls.

(G) Enrichment analysis of the differentially methylated regions (DMR) that have higher methylation (hyper_DMR) or lower methylation level (hypo_DMR) in T28KO (U1-9) naïve hESCs relative to Ctrl.

(H) TRIM28 binding of HERVH family members first defined as being up-regulated/down-regulated or unchanged in T28KO (U1-9) naïve hESCs relative to Ctrl. Notably, the up-regulated HERVH family members in T28KO naïve hESCs exhibit two peaks of TRIM28 binding, whereas the other groups do not.

Supplemental experimental procedures

Primed and naïve hESC culture

The primed hESC media was composed of 20% knockout serum replacement (KSR) (GIBCO), 100μM L-Glutamine (GIBCO), 1x MEM Non-Essential Amino Acids (NEAA) (GIBCO), 55μM 2-Mercaptoethanol (GIBCO), 10ng/mL recombinant human FGF basic (R&D systems), 1x Penicillin-Streptomycin (GIBCO), and 50ng/mL primocin (InvivoGen) in DMEM/F12 media (GIBCO). The primed hESCs were split by 1mg/ml Collagenase type IV (GIBCO) and maintained routinely on mitomycin C (MMC)-inactivated mouse embryonic fibroblasts (MEFs). Naïve hESCs were established by reverting the primed hESCs with 5iLAF media as described before (Theunissen et al., 2014). The composition of 5iLAF media was: DMEM/F-12 (Life Technologies) and Neurobasal (Life Technologies), with 1x N2 supplement (Life Technologies), 1x B27 supplement (Life Technologies), 8ng/mL bFGF (Peprotech), 1% nonessential amino acids (Life Technologies), 1mM GlutaMAX (Life Technologies), penicillin- streptomycin (Life Technologies), 0.1mM beta-mercaptoethanol (Life Technologies), 50μg/mL BSA (Sigma), 1μM PD0325901 (Stemgent), 1μM IM-12 (Enzo), 0.5μM SB590885 (R&D Systems), 1μM WH-4-023 (A Chemtek), 10μM Y-27632 (Stemgent), 20ng/mL Activin A (Peprotech), 20ng/mL rhLIF (Millipore) and 0.5% KSR (Life Technologies). The basic procedure was: primed cells were split to single cells by treatment with 0.05% Trypsin-EDTA (Life Technologies) for 3 minutes at 37ºC, after dissociation to single cells in hESC media+ 10uM ROCKi Y-27632 (Stemgent), cells were forced through a 40μm cell strainer, and 200K single cells were seeded onto MMC-treated feeders in hESC media+ROCKi; two days later, the media was changed to 5iLAF and replaced with fresh media every day until day 10, and the cells were then split again with Accutase (Innovative Cell Technologies), 200K cells were then seeded onto new feeders, colonies would start to form at subsequent passages, and naïve hESCs were then maintained in 5iLAF media.

Immunofluorescence staining

We performed immunofluorescence staining according to previous protocol (Gkountela et al., 2015; Li et al., 2015). Briefly cells were cultured on chamber slides, when reached 80%-90% confluency, cells were fixed with 4% paraformaldehyde in PBS for 10 minutes, washed with PBS containing 0.1% Tween 20 and permeabilized with PBS containing Triton-X100 for 10 min. Samples were blocked with 10% donkey serum for 30 minutes. Samples were incubated with goat-anti-OCT4 (Santa Cruze Biotechnology, sc-8628, 1:100), or Rabbit-anti-TRIM28 (Abcam, ab10483) overnight at 4ºC. The secondary antibodies were donkey-anti-rabbit-488 (Jackson ImmunoResearch Laboratories, 711-545-152) and donkey-anti-goat-594 (Jackson ImmunoResearch Laboratories, 705-586-147). DAPI was included in the mounting medium for indicating the nucleus.

Western blot

Cells were harvested and resuspended in 1x Laemmli buffer, and denatured for 10-15 minutes at 95ºC. Appropriate amount of samples were run on a NuPAGE 4-12% Bis-Tris gel (Invitrogen), gels were transferred at 200mA for 1.5 hours, and blots were blocked with 1xOdyssey blocking buffer overnight (Licor), followed by primary antibody incubation for 2 hours at room temperature, then secondary antibody incubation for 45 minutes at room temperature. The blots were then dried and imaged on Odyssey Infrared Imager (Licor). The primary antibodies using in this work are: rabbit-anti-TRIM28 (Abcam ab10483, 1:2000), mouse-anti-bACTIN (santa cruz sc47778, 1:1000), the secondary antibodies are 800CW-goat-anti-rabbit and 800CW-goat-anti-mouse (Licor, 1:20,000).

Naïve hESC flow cytometry analysis

Day 7 naïve hESCs were dissociated with 0.05% Trypsin-EDTA for 3-5 minutes at 37℃. The dissociated cells were stained with antibodies which were PE conjugated with TRA1-85 (R&D Systems) and SSEA4 conjugated with APC (R&D Systems) for at least 1hr on ice, then cells were washed with FACS buffer (1% BSA in PBS) once and resuspended in FACS buffer with DAPI (BD Pharmingen), finally cells were passed through 40μm cell strainer (Fisher scientific) and ready for flow cytometry analysis.

EB differentiation

Confluent hESCs were treated with Collagenase type IV (GIBCO) for 45-60 minutes at 37ºC, until the colonies start detaching from feeders when the plate was tapped, cells were collected and allowed to settle by gravity, then the supernant was carefully removed. The cells were resuspended with mTeSR (Stemcell Technologies) +10uM ROCKi (Stemgent) and then transfer to 6-well low attachment plate (Corning). 24 hours later media was changed to Differentiation Media which is composed of

DMEM/F12 media (Invitrogen/GIBCO), 20% ES FBS (Hyclone), 100µM L-Glutamine (GIBCO), 1x MEM Non-Essential Amino Acids (NEAA) (GIBCO), and 55µM 2-Mercaptoethanol (GIBCO), change media every 48 hours, collect cells at day 2 and day 5 for further analysis.

Real time quantitative PCR
Cell pellets were lysed in 350µL RLT buffer (QIAGEN) and RNA was extracted using RNeasy micro kit (QIAGEN, 74004). cDNA was synthesized using SuperScript® II Reverse Transcriptase (Invitrogen, 18064-014). Real time quantitative PCR was performed using TaqMan® Universal PCR Master Mix (Applied Biosystems, 4304437) and the expression levels of genes-of-interest were normalized to the expression of the housekeeping gene GAPDH. The Taqman probes are: GAPDH (Applied Biosystems, Hs99999905_m1), H19 (Applied Biosystems, Hs00399294_g1), MEG3 (Applied Biosystems, Hs00292028_m1).

ChIP-qPCR
ChIP assays were performed with approximately 1 million cells per experiment, based on a previously described protocol with slight modifications (Boyer et al., 2005). Briefly, cells were crosslinked with 1% formaldehyde for 10 mins at room temperature and formaldehyde was quenched by glycine to a final concentration of 0.125 M. Chromatin was sonicated to an average size of 0.5–2 kb by Bioruptor (Diagenode). A total of 3 mg of antibody was added to the sonicated chromatin and incubated overnight at 4ºC. 10% of chromatin used for each ChIP reaction was kept as an input DNA. Subsequently, 60µL of protein A magnetic Dynabeads (Invitrogen) were added to the ChIP reactions and incubated for two additional hours at 4ºC. Magnetic beads were washed and chromatin eluted, followed by reverse crosslinking and DNA purification. Resultant ChIP DNA was dissolved in elution buffer from Qiagen PCR Purification Kit.
For ChIP-qPCR, 2 sets of primers were designed for each locus, qPCR was performed as triple technical replicates, results were determined as percentage of input. Antibodies used for ChIP: TRIM28 (Abcam, ab10483), H3K9me3 (Abcam, ab8898). Primer sets for ChIP-qPCR: Primer set 1 (H19 locus): H19_1_Frw – CCATTTCAGGGTGTGGGGTG, H19_2_Rev – TTGCTGCTTGGACTAGGGTC. Primer set 3 (H19 locus): H19_3_Frw – GGTGTGCTGGGATGCTTTAAC, H19_4_Rev –CTTGACAATCAGCCTGGGTG. Primer set 11 (Meg3 promoter): MEG3_11_Frw – CCATACATGTGCAGGGTGAGAC, MEG3_12_Rev – GTCCTGGGTGTTCCTGAGGTATC. Primer set 13 (Meg3 promoter): MEG3_13_Frw– GACTAAGCCATACATGTGCAGG, MEG3_14_Rev – CCTGAGTGAGCTCTAGGCATTG.

Cardiomyocyte differentiation
Confluent hESCs were enzymatically detached and transferred into low attachment dish (Corning) with ESC Culture Medium as described earlier. Cells were cultured in serum-free cardiac differentiation medium which is composed of IMDM containing 1% MEM nonessential amino acid solution, 1% penicillinstreptomycin, 2 mM L-glutamine, 0.5 mM L-carnitine (Sigma-Aldrich), 0.001% 2-mercaptoethanol, and 0.4% human serum albumin (Sigma-Aldrich), with 4 mM CHIR (Axon) and 2 mM BIO (Calbiochem). On days 3–9, 10 mM KY02111 and/or other WNT inhibitors (XAV939 and/or IWP-2) were added to cell cultures, and the medium was changed every 2 days. On day 14, the cardiac colonies were collected for further analysis.

Bioinformatics analysis

RNA-seq analysis
mRNA reads were aligned to the human reference genome (hg19) using Tophat2 (Kim et al., 2013). The gene annotation was downloaded from iGenome (iGenome). The expression counts of genes were generated using HTSeq-count (Anders et al., 2015). To ensure the statistical power, genes have to be expressed (reads > 0) in at least 2 out of 3 replicates in both primed hESCs and naïve hESCs. To exclude the low expression genes, the most expressed replicate had to have a minimum of 10 reads. The annotation of TE was downloaded from (Theunissen et al., 2016) The expression counts of TEs were generated using multiBamCov from BEDtools (Quinlan and Hall, 2010). A TE was considered expressed, if it was expressed in one or more samples (RPKM>0). To exclude the low expression TEs, the most expressed replicate had to have a minimum of 10 reads (after normalizing for sequencing depth). Normalization for sequencing depth and differential gene/TE-expression analysis was performed using edgeR (Robinson et al., 2010). Genes or TEs with ≥2-fold difference in expression and FDR <5% were deemed differentially expressed.

Profiling genome-wide DNA methylation

Bisulfite converted reads were aligned to the human reference genome (hg19) using BS-Seeker 2 (Guo et al., 2013). Genome-wide DNA methylation profiles were generated by determining methylation levels for each cytosine in the genome. Since bisulfite treatment converts unmethylated cytosines (Cs) to thymines (Ts) after PCR amplification, the methylation level at each cytosine was determined as #C/(#C+#T), where #C represents the number of methylated reads and #T represents the number of unmethylated reads. The methylation level per cytosine serves as an estimate of the percentage of cells that have a methylated cytosine at a specific cytosine site. We only included cytosines that were covered by at least three reads. To identify differentially methylated regions (DMRs), a non-overlapping tiling window approach was used for genome-wide screening, where genomic regions of 500 bp containing ≥ 4 CpG sites in all compared samples were surveyed. A region is deemed differentially methylated if it shows ≥ 50% difference in average methylation level and a t-test p-value < 5%. A gene is considered differentially methylated (DMG) if it contains one or more DMRs in promoter or gene body.

ATAC-seq analysis

ATAC-seq reads were mapped to the human reference genome (hg19) using Bowtie2 (Langmead and Salzberg, 2012). Reads mapped to mitochondrial DNA were excluded from the analysis. For each sample the peaks of ATAC-seq reads are considered accessible regions, which were identified using MACS2 (Feng et al., 2012). To determine differential abundance of peaks between samples (i.e. regions that switch accessibility), the reads are normalized to 1X using deeptools (Ramírez et al., 2016). The resultant peak was considered differentially accessible if the foldchange of abundance at the peak between T28KO and Ctrl was ≥ 8, and a t-test p value < 5%.

ChIP-seq analysis

Raw reads of TRIM28 ChIP-seq downloaded from GSE84382 were mapped to the human reference genome (hg19) using Bowtie2 (Langmead and Salzberg, 2012). Peak calling was performed using MACS2 (Feng et al., 2012).

Supplemental References

Anders, S., Pyl, P.T., and Huber, W. (2015). HTSeq—a Python framework to work with high-throughput sequencing data. Bioinformatics *31*, 166-169.

Boyer, L.A., Lee, T.I., Cole, M.F., Johnstone, S.E., Levine, S.S., Zucker, J.P., Guenther, M.G., Kumar, R.M., Murray, H.L., Jenner, R.G.*, et al.* (2005). Core Transcriptional Regulatory Circuitry in Human Embryonic Stem Cells. Cell *122*, 947-956.

Feng, J., Liu, T., Qin, B., Zhang, Y., and Liu, X.S. (2012). Identifying ChIP-seq enrichment using MACS. Nat. Protocols *7*, 1728-1740.

Gkountela, S., Zhang, Kelvin X., Shafiq, Tiasha A., Liao, W.-W., Hargan-Calvopiña, J., Chen, P.-Y., and Clark, Amander T. (2015). DNA Demethylation Dynamics in the Human Prenatal Germline. Cell *161*, 1425-1436.

Guo, W., Fiziev, P., Yan, W., Cokus, S., Sun, X., Zhang, M.Q., Chen, P.-Y., and Pellegrini, M. (2013). BS-Seeker2: a versatile aligning pipeline for bisulfite sequencing data. BMC Genomics *14*, 774.

Kim, D., Pertea, G., Trapnell, C., Pimentel, H., Kelley, R., and Salzberg, S.L. (2013). TopHat2: accurate alignment of transcriptomes in the presence of insertions, deletions and gene fusions. Genome Biology *14*, R36.

Langmead, B., and Salzberg, S.L. (2012). Fast gapped-read alignment with Bowtie 2. Nat Meth *9*, 357-359.

Li, Z., Yu, J., Hosohama, L., Nee, K., Gkountela, S., Chaudhari, S., Cass, A.A., Xiao, X., and Clark, A.T. (2015). The Sm protein methyltransferase PRMT5 is not required for primordial germ cell specification in mice. The EMBO Journal *34*, 748-758.

Pastor, William A., Chen, D., Liu, W., Kim, R., Sahakyan, A., Lukianchikov, A., Plath, K., Jacobsen, Steven E., and Clark, Amander T. (2016). Naive Human Pluripotent Cells Feature a Methylation Landscape Devoid of Blastocyst or Germline Memory. Cell Stem Cell *18*, 323-329.

Quinlan, A.R., and Hall, I.M. (2010). BEDTools: a flexible suite of utilities for comparing genomic features. Bioinformatics *26*, 841-842.

Ramírez, F., Ryan, D.P., Grüning, B., Bhardwaj, V., Kilpert, F., Richter, A.S., Heyne, S., Dündar, F., and Manke, T. (2016). deepTools2: a next generation web server for deep-sequencing data analysis. Nucleic Acids Research *44*, W160-W165.

Robinson, M.D., McCarthy, D.J., and Smyth, G.K. (2010). edgeR: a Bioconductor package for differential expression analysis of digital gene expression data. Bioinformatics *26*, 139-140.

Theunissen, Thorold W., Friedli, M., He, Y., Planet, E., O'Neil, Ryan C., Markoulaki, S., Pontis, J., Wang, H., Iouranova, A., Imbeault, M.*, et al.* (2016). Molecular Criteria for Defining the Naive Human Pluripotent State. Cell Stem Cell.
