# Supplementary material for: TRIM28-Regulated Transposon Repression Is Required for Human Germline Competency and Not Primed or Naive Human Pluripotency
Source: Stem Cell Reports. 2017 Dec 28;10(1):243–56. doi: 10.1016/j.stemcr.2017.11.020 (PMC5768987; doi:10.1016/j.stemcr.2017.11.020)
Supplement: Document S2. Article plus Supplemental Information [file mmc6.pdf]

Stem Cell Reports
Article
OPEN ACCESS

TRIM28-Regulated Transposon Repression Is Required for Human Germline Competency and Not Primed or Naive Human Pluripotency

Yu Tao,[1,6] Ming-Ren Yen,[5,6] Tsotne Chitiashvili,[1,3] Haruko Nakano,[1] Rachel Kim,[2] Linzi Hosohama,[1] Yao Chang Tan,[1] Atsushi Nakano,[1,2,3,4] Pao-Yang Chen,[5,*] and Amander T. Clark[1,2,3,4,*]
[1]Department of Molecular Cell and Developmental Biology
[2]Eli and Edythe Broad Center of Regenerative Medicine and Stem Cell Research
[3]Molecular Biology Institute
[4]Jonsson Comprehensive Cancer Center
University of California, Los Angeles, Los Angeles, CA 90095, USA
[5]Institute of Plant and Microbial Biology, Academia Sinica, Taipei 11529, Taiwan
[6]Co-first author
*Correspondence: paoyang@gate.sinica.edu.tw (P.-Y.C.), clarka@ucla.edu (A.T.C.)
https://doi.org/10.1016/j.stemcr.2017.11.020

SUMMARY

Transition from primed to naive pluripotency is associated with dynamic changes in transposable element (TE) expression and demethylation of imprinting control regions (ICRs). In mouse, ICR methylation and TE expression are each regulated by TRIM28; however, the role of TRIM28 in humans is less clear. Here, we show that a null mutation in *TRIM28* causes significant alterations in TE expression in both the naive and primed states of human pluripotency, and phenotypically this has limited effects on self-renewal, instead causing a loss of germline competency. Furthermore, we discovered that TRIM28 regulates paternal ICR methylation and chromatin accessibility in the primed state, with no effects on maternal ICRs. Taken together, our study shows that abnormal TE expression is tolerated by self-renewing human pluripotent cells, whereas germline competency is not.

INTRODUCTION

Most studies on the mechanisms that regulate human pluripotency have focused on protein-coding genes, which constitute less than 5% of the human genome. In contrast, TEs, which account for nearly half the human genome, have received significantly less attention. Recent reports have shown that TEs are dynamically expressed in human germline cells, the naive and primed states of human pluripotency, human pre-implantation embryos, and human germ cell tumors (Göke et al., 2015; Grow et al., 2015; Herbst et al., 1996; Lu et al., 2014; Theunissen et al., 2016; Wang et al., 2014). Yet the mechanisms that regulate the dynamic expression of TEs in human pluripotency and human germline development are not well understood.

One of the most dynamically expressed families of TEs in human pre-implantation embryos are long terminal repeat (LTR) retrotransposons, which constitute about 8% of the human genome (Cordaux and Batzer, 2009). Notably, full-length human-specific LTR5 (LTR5_HS) human endogenous retroviral K (HERVK) TEs are expressed exclusively in 8-cell, morula, and pre-implantation human epiblast cells as well as in germ cell tumors (Grow et al., 2015; Herbst et al., 1996). In contrast, the primate-specific LTR7-HERVHs are expressed throughout human pre-implantation embryo development as well as in primed human embryonic stem cells (hESCs), but are repressed when primed hESCs are converted to the naive state (Theunissen et al., 2016). LTR5_HS TEs regulate viral infection in human pluripotent stem cells, whereas LTR7-HERVHs regulate primed pluripotent stem cell self-renewal (Göke et al., 2015; Grow et al., 2015; Lu et al., 2014; Wang et al., 2014).

Dynamic TE expression is not restricted to human embryos. In the mouse embryo, murine endogenous retrovirus L (MuERV-L) is expressed at the 2-cell and 8-cell stage of mouse embryo development, whereas intracisternal A particles (IAPs) are expressed in mouse oocytes, cleavage embryos, and blastocysts (Svoboda et al., 2004). Long interspersed nuclear element 1 (LINE1), a non-LTR TE, is expressed during mouse zygotic genome activation where it functions to enable chromatin accessibility (Fadloun et al., 2013; Jachowicz et al., 2017; Rowe et al., 2010). Therefore TE expression in germline competent pluripotent cells in both mouse and human is a fundamental requirement for pluripotent cell biology.

Although regulation of TE expression in human embryos and pluripotent stem cells is not well known, one of the major mechanisms responsible for regulating TE expression in mouse embryos is *Trim28* (also named Kap1 and Tif1b) (Schultz et al., 2001, 2002; Wolf and Goff, 2007; Zuo et al., 2012). In mouse, a zygotic knockout of *Trim28* causes embryonic lethality shortly after implantation (Cammas et al., 2000), whereas a maternal mouse knockout causes variable epigenetic instability at imprinting control regions (ICRs), and no live births (Messerschmidt et al., 2012). In naive mouse ESCs, *Trim28* is essential for repression of IAPs, as well as mouse ESC self-renewal and survival (Rowe et al., 2010), whereas in primed hESCs, a short-term

This is an open access article under the CC BY license (http://creativecommons.org/licenses/by/4.0/).

knockdown of *TRIM28* leads to HERV derepression (Turelli et al., 2014); however, the role of TRIM28 in the basic properties of human primed or naive pluripotency is not known.

In the current study we report the generation of *TRIM28* null mutations in primed and naive hESCs using clustered regularly interspaced short palindromic repeats (CRISPR)/CRISPR-associated protein nuclease (Cas9) technology (Cong et al., 2013). We show that a null mutation in *TRIM28* is compatible with both primed and naive human self-renewal, despite massive derepression of TEs. We demonstrate that a null mutation in *TRIM28* leads to loss of human germline competency from primed hESCs, indicating that it is the ability to differentiate into the germline, not pluripotent self-renewal per se, that is particularly sensitive to loss of TRIM28 in humans.

RESULTS

TRIM28 Is Not Required for Primed hESC Self-Renewal

To evaluate the function of TRIM28 in primed hESCs, we generated targeted deletions of *TRIM28* in the genome using CRISPR/Cas9 in the karyotypically normal 46XX UCLA1 and 46XY UCLA6 hESC lines (Diaz Perez et al., 2012). To achieve this, we designed paired guide RNAs (gRNAs) that targeted exon 4 and exon 11 of human *TRIM28* (Figure S1A). Following co-electroporation of plasmids expressing the gRNAs and Cas9, individual clones were picked and genotyped. A total of 112 UCLA1 and 48 UCLA6 clones were screened, and we identified two potential homozygous mutant hESC clones in UCLA1 that we called TRIM28 knockout (T28KO) UCLA1-9 (U1-9) and UCLA1-11 (U1-11) and one homozygous mutant in UCLA6 called T28KO U6. Control (Ctrl) clones in UCLA1 and UCLA6 hESC lines were created by electroporating Cas9 without gRNAs. To identify the precise mutation, we performed PCR followed by cloning and Sanger sequencing of individual alleles from the T28KO genome (Figure S1A). Western blot analysis was used to confirm that the TRIM28 protein was not expressed in any of the three sublines of T28KO hESCs relative to Ctrls (Figure 1A). Therefore, we conclude that our gene-editing approach creates null *TRIM28* mutations in primed hESCs.

Unlike in mouse where a null mutation in *Trim28* leads to defects in mouse ESC self-renewal, T28KO hESC lines were capable of self-renewal, maintaining a morphology of round, tightly packed colonies that were indistinguishable from Ctrl (Figure S1B). Analysis of cell number after passaging T28KO and Ctrl UCLA1 sublines revealed that on average T28KO hESC cultures had more cells on day 7 after plating compared with the Ctrl (Figure 1B), suggesting that T28KO hESCs grow faster. We also discovered that T28KO hESCs are karyotypically normal, even after more than ten passages of culture (Figure S1C). Furthermore, T28KO hESCs expressed typical markers of self-renewal and pluripotency including OCT4 (Figure 1C), and were capable of teratoma formation when transplanted into immunocompromised mice (Figure 1D). Although the teratomas contained cell types from the three somatic lineages based on histology, the T28KO teratomas were darkly pigmented upon necropsy, and histological analysis indicated an abundance of highly pigmented somatic cells (Figure S2A). These pigmented cells were rarely identified in teratomas derived from Ctrl hESCs. Therefore, our data indicate that a null mutation in TRIM28 has no effect on self-renewal or pluripotency in primed conditions; instead, it is associated with faster growth and a preference for generating pigmented somatic cells in teratoma assays together with cell types representing the major somatic embryonic layers.

TRIM28 Is Required for Germline Competency

Although the teratoma assay is a non-quantitative method for assessing the ability to differentiate into somatic cells, it provides no clues as to whether hESC lines are germline competent. To address this, we differentiated Ctrl and T28KO hESCs into human primordial germ cell-like cells (hPGCLCs) following the protocol established by Sasaki et al. (2015). In this approach, hPGCLCs are differentiated through a two-step protocol involving 24 hr of adherent differentiation to create incipient mesoderm-like cells (iMeLCs), followed by aggregate differentiation in 96-well plates to create aggregates containing hPGCLCs (Figure 2A). To identify hPGCLCs in the aggregates, we used fluorescence-activated cell sorting (FACS) to isolate hPGCLCs expressing the cell surface receptors integrin alpha 6 (ITGA6) and epithelial cell adhesion molecule (EPCAM) (Figure 2B). Our results showed that Ctrl UCLA1 and UCLA6 hESCs had a clearly defined ITGA6/EPCAM double-positive hPGCLC population at day 4 of differentiation (Figure 2B), whereas all three T28KO sublines did not produce hPGCLCs (Figure 2B); we quantified the percentages of the double-positive populations (Figure 2C). Therefore, *TRIM28* null hESCs cultured in the primed state of pluripotency have lost germline competency.

Given that T28KO hESCs are germline incompetent, we hypothesized that there might be also somatic lineage bias, particularly given the pigmented teratomas. To address this, we performed spontaneous embryoid body (EB) differentiation and real-time PCR to examine ectoderm, mesoderm, and endoderm induction at days 2 and 5 of EB differentiation (Figures S2B–S2D). We discovered some gene-specific differences between T28KO and Ctrl EBs, but collectively ectoderm (*SOX1*, *PAX6*, and *ZIC1*), endoderm (*SOX17*, *GATA6*, and *FOXA1*), and

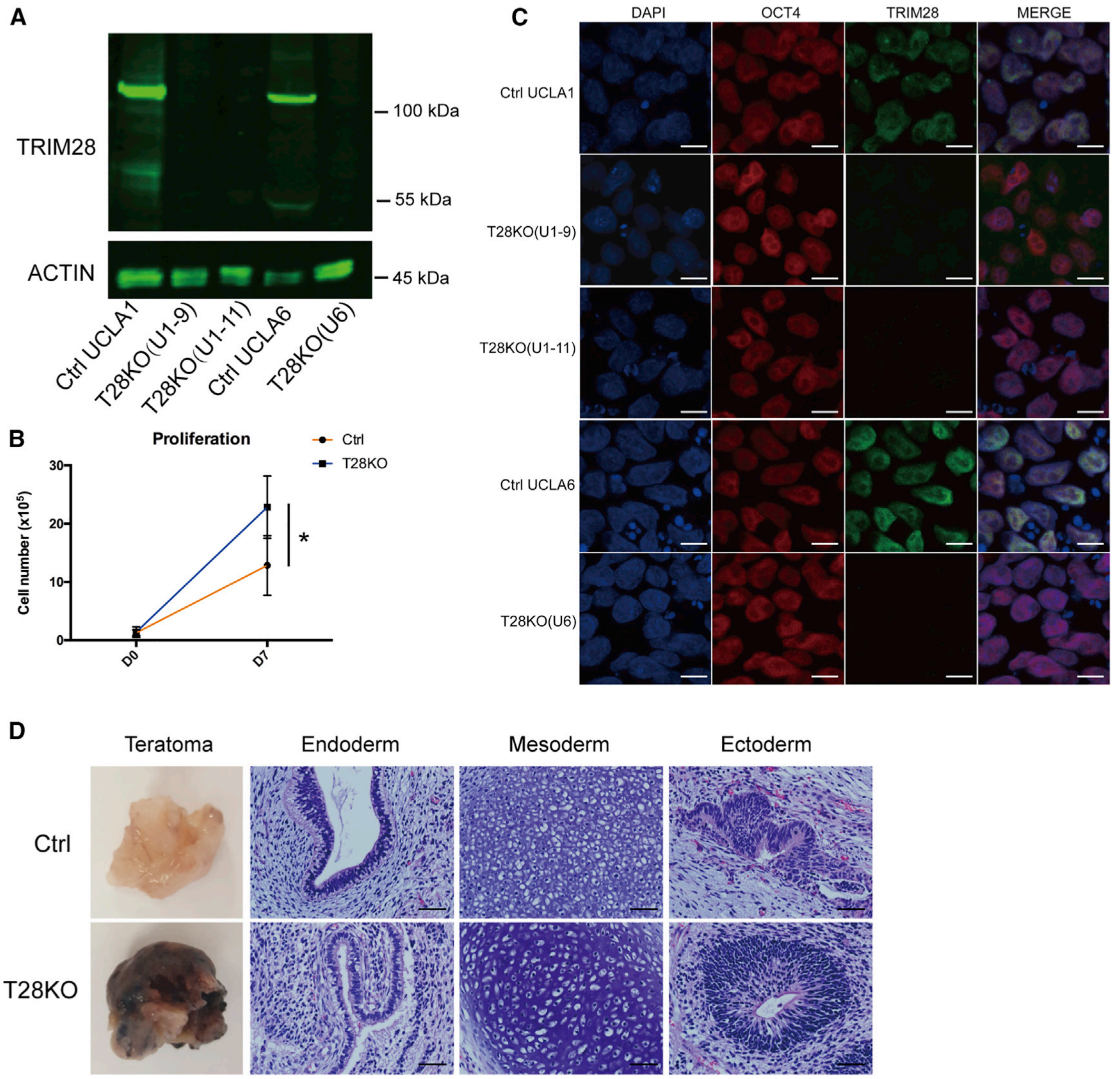

Figure 1. TRIM28 Is Not Required for Primed hESC Self-Renewal or Pluripotency

(A) Western blot to detect TRIM28 in control UCLA1 (Ctrl UCLA1), TRIM28KO #9 and #11 in UCLA1 (T28KO(U1-9) and T28KO(U1-11)), control UCLA6 (Ctrl UCLA6), and TRIM28KO in UCLA6 (T28KO(U6)) primed hESCs. The top band represents the expected full-length TRIM28 protein (110 kDa). ACTIN is an internal loading control.

(B) Average cell counts over 7 days of control (Ctrl UCLA1) and T28KO (T28KO(U1-9)) primed hESC culture (n = 3 independent experiments), $^{*}p < 0.05$. Error bars represent SD.

(C) Representative immunofluorescence for OCT4 (red), TRIM28 (green), and nuclei (DAPI). Scale bar, 10 μm.

(D) Representative images of H&E-stained teratoma sections from control (Ctrl UCLA1) and T28KO (T28KO(U1-9)) hESCs (n = 6 teratomas). Scale bar, 50 μm.

See also Figure S1.

mesoderm (*MIXL1* and *TBX3*) genes were induced into T28KO EBs (Figures S2B–S2D). One transcription factor, *HAND1*, was consistently lower in T28KO EBs at days 2 and 5 of EB differentiation experiments relative to Ctrls, suggesting that mesoderm cell lineage differentiation requiring *HAND1* (for example, the differentiation of cardiomyocytes) could be compromised. To address this, we performed directed differentiation into cardiomyocytes (CMs), and showed that the percentage of CMs was similar between Ctrl and T28KO at day 14 of differentiation (Figures S2E and S2F). However, when normalized to the total number of undifferentiating hESCs used to initiate CM differentiation, the ratio of CMs generated per input of hESCs was significantly lower in T28KOs (Figure S2G). Taken together, a null mutation in TRIM28 had almost no effect on the basic properties of hESC

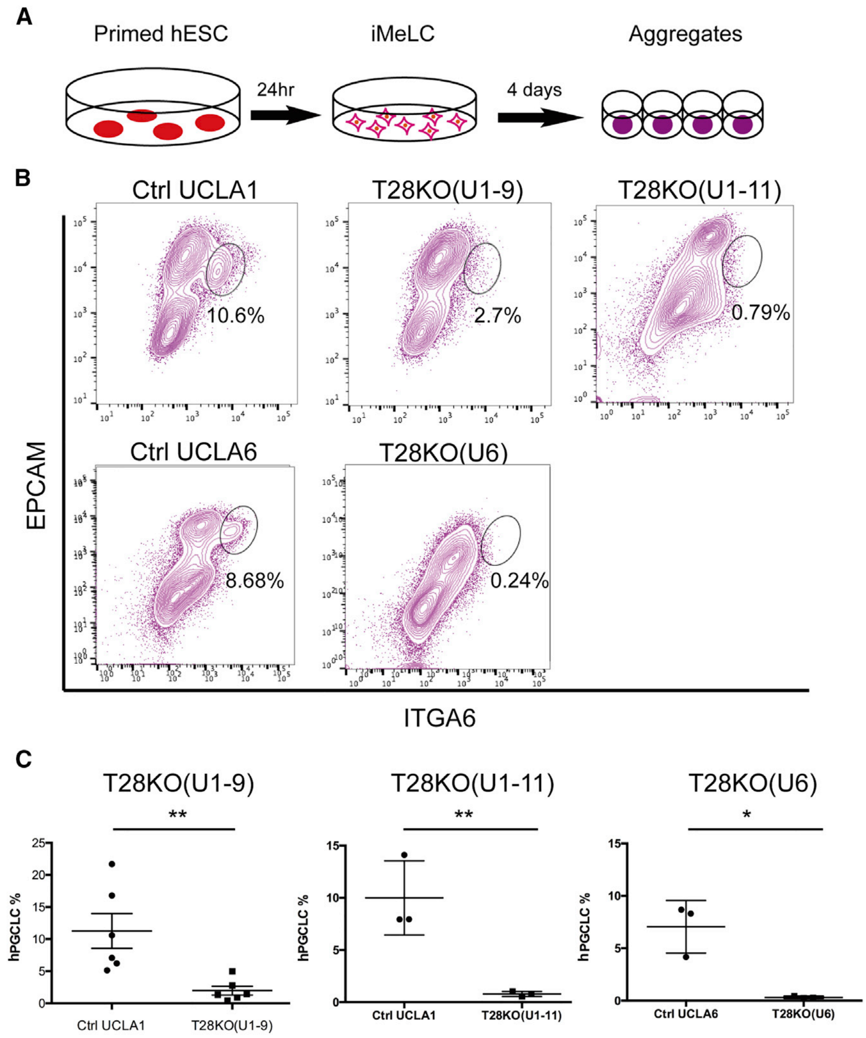

Figure 2. TRIM28KO hESCs Are Germline Incompetent

(A) Schematic description of hPGCLC differentiation.

(B) Representative flow-cytometry plots of day-4 aggregates in Ctrl UCLA1, T28KO(U1-9), T28KO(U1-11), Ctrl UCLA6, and T28KO(U6). The hPGCLC population is circled.

(C) Average percentage of hPGCLCs at day 4 of aggregate differentiation in paired experiments of T28KO(U1-9) (n = 6 independent experiments) with Ctrl UCLA1 (n = 6 independent experiments), T28KO(U1-11) (n = 3 independent experiments) with Ctrl UCLA1 (n = 3 independent experiments), and T28KO(U6) (n = 3 independent experiments) with Ctrl UCLA6 (n = 3 independent experiments), $^{**}p < 0.01$, $^{*}p < 0.05$. Error bars represent SD.

See also Figure S2.

self-renewal, and instead affects germline competency and the ability to maintain certain cell types derived from mesoderm, most notably CMs.

TRIM28 Is Required to Repress HERVHs, SVAs, and ZNFs in Primed hESCs

Previous studies found that TRIM28 is bound to the chromatin of primed hESCs, and is specifically enriched at HERVs, LTRs, and SVA (SINE-R, VNTR, and Alu) TE subfamilies (Theunissen et al., 2016 and Figure S3A). To examine how a null mutation in TRIM28 affects TE expression, we performed RNA sequencing (RNA-seq) and discovered that T28KO hESCs had an abundance (10,949) of differentially expressed TEs (DETEs) with a smaller number (670) of differentially expressed genes (DEGs) relative to Ctrl cells (Figure 3A; Tables S1 and S2). One of the DEGs was *TRIM28* (Figure S3B). Enrichment analysis of the DETEs revealed that this group was highly enriched in HERV, LTR, and SVAs as anticipated from chromatin immunoprecipitation sequencing (ChIP-seq) of TRIM28 in wild-type primed hESCs (Figure 3B).

Given the dynamic expression of HERVH and HERVK in human pre-implantation embryos and the re-expression of SVA when primed hESCs are switched to the naive state, we focused specifically on these subfamilies. Using the ChIP-seq dataset of Theunissen et al. (2016), we identified expressed HERVH, HERVK, and SVA TEs that were previously reported as being bound by TRIM28 in primed hESCs (Theunissen et al., 2016). Of the 1,400 expressed HERVH sequences in our RNA-seq dataset (where expression was >10 reads in at least one library) only 81 were bound by TRIM28, and on average these TEs were derepressed in T28KO hESCs relative to Ctrls (Figures 3C and S3C). In contrast the 169 TRIM28-bound HERVKs were unchanged (Figures 3D and S3D). Similar to HERVH subfamily members, the 293 TRIM28-bound SVAs were also significantly upregulated in T28KOs relative to Ctrls (Figure 3E). Therefore, TRIM28 had subfamily-specific effects on TE

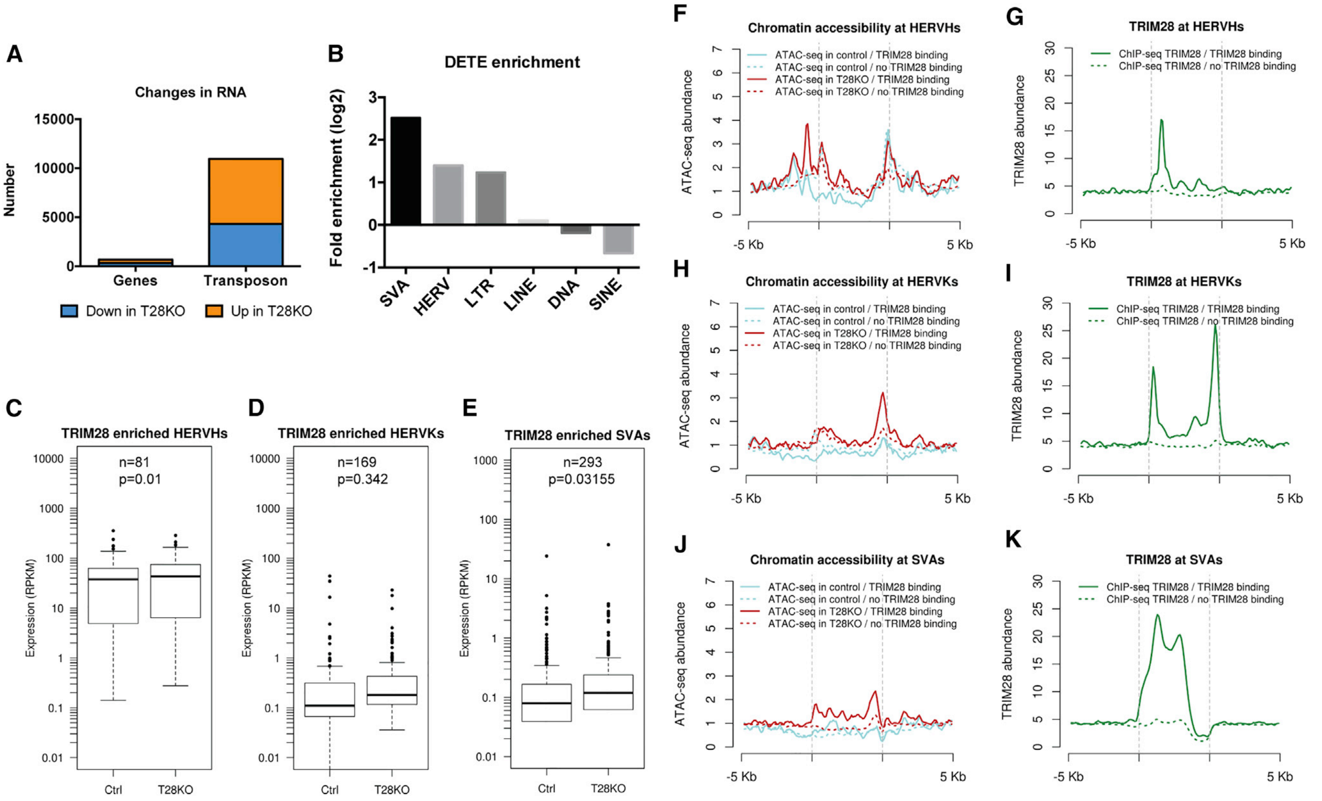

Figure 3. TRIM28 Is Required to Repress HERVH and SVA Family Members in Primed hESCs

(A) RNA-seq was used to detect DEG (n = 670) and DETE (n = 10,949) between Ctrl and T28KO(U1-9) primed hESCs.

(B) Enrichment analysis of the DETE between Ctrl and T28KO(U1-9) primed hESCs in TE subfamilies. Fold enrichment is represented as $\log_2$(T28KO/Ctrl).

(C–E) Box plots of TRIM28-enriched HERVH, HERVK, and SVA expression in Ctrl and T28KO(U1-9) primed hESC. "TRIM28 enriched" denotes any expressed HERVH/HERVK/SVA that were also defined as being bound by TRIM28 in wild-type primed hESC using the ChIP-seq dataset of Theunissen et al. (2016). RPKM, reads per kilobase of transcript per million mapped reads.

(F–K) Meta-plots showing the correlations between TRIM28 binding and chromatin accessibility at HERVH, HERVK, and SVA family members in Ctrl and T28KO(U1-9) primed hESCs. The four points on the x axis show, from left to right, 5 kbp upstream of TE, 5′ end of TE, 3′ end of TE, and 5 kbp downstream of TE.

See also Figure S3.

expression in primed hESCs, most notably repressing SVAs and HERVH TEs, with limited effects on HERVK RNA expression.

To determine how a TRIM28 deletion affects chromatin accessibility, we performed an assay for transposase-accessible chromatin sequencing (ATAC-seq) and showed that chromatin accessibility was also dynamically changed in the TE subfamily members bound by TRIM28 (Figures 3F–3K). Specifically, the 5′ end of HERVH exhibited increased accessibility in T28KO hESCs relative to Ctrl cells, and these new regions of accessibility occurred on average 2 kb upstream of the TRIM28 binding site (Figure 3F). The TRIM28 bound HERVK subfamilies exhibited increased accessibility exclusively at the 3′ but not the 5′ end of the TE (Figure 3H). ChIP-seq of TRIM28 in wild-type cells shows that TRIM28 is bound to both ends of the element (Figure 3I), and this might explain the lack of HERVK expression defects in T28KO hESCs with only one of the two ends becoming more accessible. TRIM28 bound SVA elements exhibit a mild increase in accessibility across the element body, consistent with TRIM28 binding across the element body in wild-type hESCs (Figures 3J and 3K). Taken together TRIM28-bound TEs show differences in accessibility upon deletion of TRIM28.

Given that TRIM28 is mostly enriched at TEs, yet we discovered 670 DEGs (Table S1), we evaluated the correlation between DETEs and their nearest neighbor DEGs given that LTRs can function as promoters and enhancers (Rotman et al., 1986). Consistent with previous studies, our data indicate that at short ranges (within 5 kb), neighboring DEGs are highly correlated with differentially expressed LTRs (r = 0.71) or differentially expressed HERVs

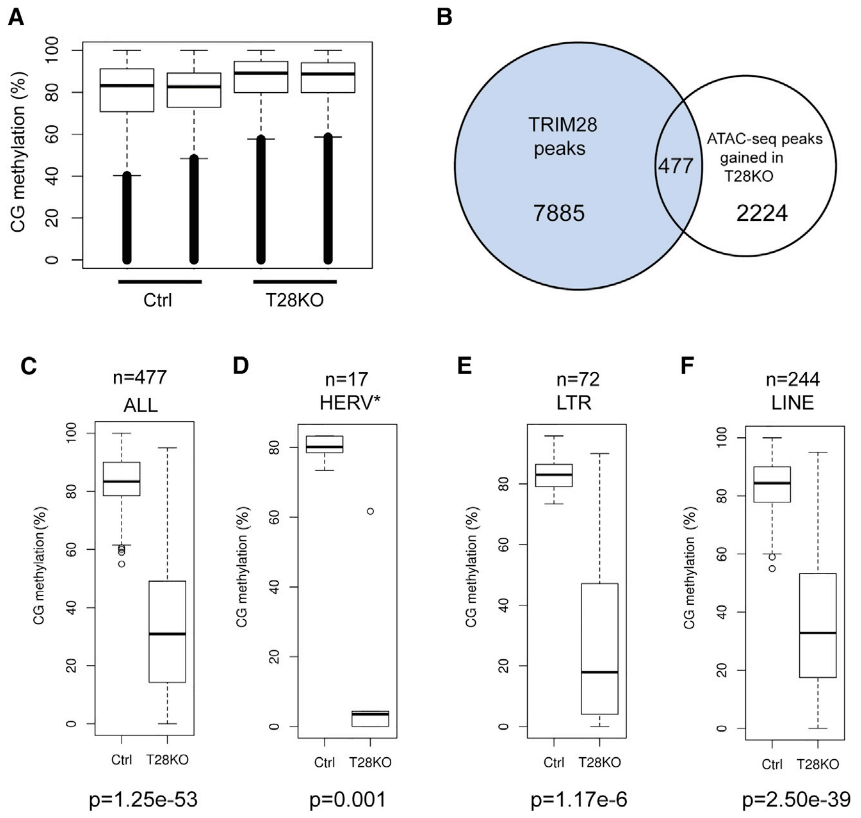

Figure 4. TRIM28 Regulates Local DNA Methylation Levels at LTRs, HERVKs, and LINEs in Primed hESCs

(A) Quantification of CpG methylation using whole-genome bisulfite sequencing (WGBS) in 528,773 common CpGs for both Ctrl and T28KO(U1-9) primed hESCs (n = 2 independent replicates).

(B) Venn diagram showing the overlap between TRIM28 binding sites in control cells using the dataset of Theunissen et al. (2016) and the emergence of new ATAC-seq peaks in T28KO(U1-9) hESCs.

(C) Percent CpG methylation levels at the 477 overlapping regions in Ctrl and T28KO(U1-9) hESCs from (B).

(D–F) Percent CpG methylation levels of HERV, LTR, and LINE from the 477 overlapping regions in Ctrl and T28KO(U1-9) hESC. Asterisk indicates that HERVs are mainly composed of HERVK.

See also Figure S4.

(r = 0.58) (Figures S3G and S3H). At increasing distances this correlation diminishes (10 kb, r = 0.35 and 20 kb, r = 0.27 for LTRs). Therefore, similar to reports in the mouse, DEGs in the T28KO hESCs may be explained by the creation of new short-range promoters/enhancers at LTRs and HERVs of neighboring genes.

To determine whether there were any functional groups significantly enriched in the DEGs, we performed gene ontology analysis. To our surprise, we discovered that the DEGs were highly enriched in genes that encoded for Krüppel-associated box domain-containing zinc-finger proteins (KRAB-ZNFs) (Figure S3I). There are 423 KRAB-ZNF genes located in 25 major clusters scattered around the human genome (Huntley et al., 2006). A subset of ZNF genes in a human erythroleukemic cell line was previously reported as being co-occupied by TRIM28, SETDB1, ATRX, and ZNF274 (Valle-García et al., 2016). ZNF274 is expressed in undifferentiated hESCs but is not differentially expressed in T28KO hESCs, so our results cannot be explained by the repression of ZNF274.

To determine whether TRIM28 is enriched in the 3′ exons of the differentially expressed ZNFs, we first examined TRIM28 binding at the 728 ZNF genes identified by Valle-García et al. (2016) in somatic cells (Figure S3J). Our data show a modest yet significant enrichment ($p = 0.002$) of TRIM28 at the 50 differentially expressed ZNFs in common with this dataset, which was mostly in the 3′ exons, as well as across the gene body (Figure S3K); the control gene set are ZNFs that show no differences in gene expression between Ctrl and T28KO hESCs ($\log_2$ fold change < 0.1). Therefore, we speculate that TRIM28 directly regulates a subset of ZNFs in primed pluripotent stem cells, and loss of TRIM28 is associated with their increased expression. Taken together, our data show that TRIM28 has subfamily-specific effects on TE expression in primed hESCs, most notably at HERVHs, LTRs, and SVAs. We also discovered that the genes regulated by TRIM28 include the ZNFs, which encode the sequence-specific binding partner KRAB-ZNF transcriptional regulators.

TRIM28 Regulates DNA Methylation and Chromatin States at LTRs, HERVKs, and LINEs in Primed hESCs

Previous studies have identified a complex relationship between DNA methylation and TRIM28 in the mouse embryo (Quenneville et al., 2012; Schultz et al., 2002; Zuo et al., 2012). Here, we examined how a null mutation in TRIM28 affects DNA methylation in primed hESCs. To achieve this, we performed whole-genome bisulfite sequencing (WGBS) of T28KO and Ctrl hESCs, and showed that global DNA methylation levels at CpGs sites remain high at >80% (Figure 4A). To examine DNA methylation levels at regions specifically bound by TRIM28, we first

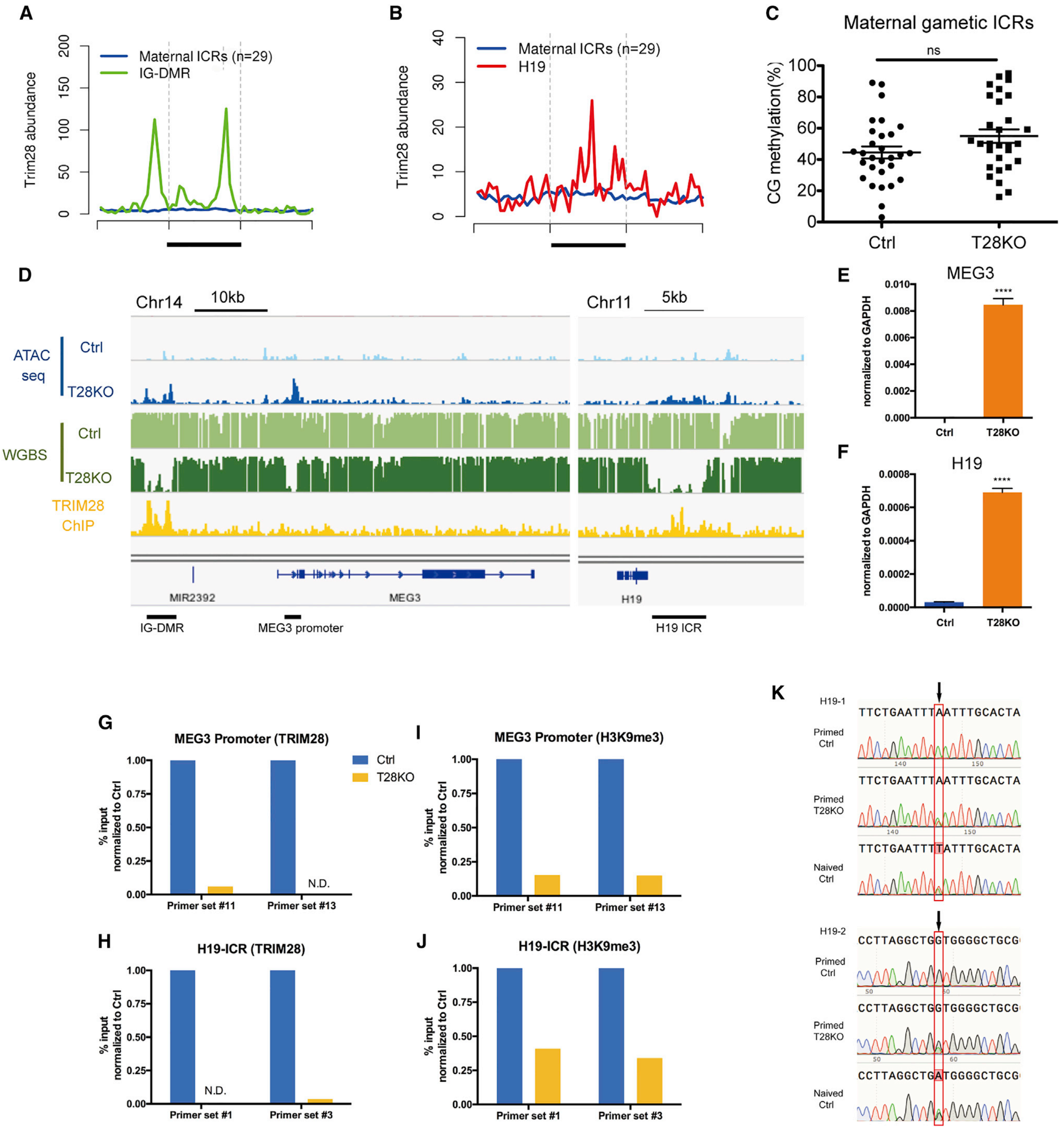

Figure 5. TRIM28 Is Required to Maintain DNA Methylation at Paternal but Not Maternal ICRs in Primed hESCs

(A and B) TRIM28 binding at the IG-DMR and the H19-ICR relative to the average TRIM28 binding at 29 maternal ICRs as a control. ICR is underlined.

(C) Percent CpG methylation levels at maternal imprinted ICRs in Ctrl and T28KO(U1-9) primed hESCs (n = 29 maternal ICRs), ns, not significant. Error bars represent SD.

(D) Browser view of ATAC-seq (blue), WGBS (green), and TRIM28 ChIP-seq (yellow) at the IG-DMR ICR, the MEG3 promoter, and the H19 ICR.

(legend continued on next page)

identified all the TRIM28 bound regions that show increased accessibility in the T28KO hESCs (Figure 4B). Critically we discovered that only a small fraction of TRIM28 bound regions (477/7,885; 6%) exhibit a significant increase in chromatin accessibility (fold change $\geq$ 8, $p < 0.05$) relative to Ctrl cells. Intriguingly, even though the 477 TRIM28 bound regions had increased accessibility, we discovered a more significant effect on the regions not bound by TRIM28. Specifically, we show that 2,224 new ATAC-seq peaks are gained in the T28KO hESCs relative to Ctrl (Figure 4B). In the current analysis, we focused on the 477 regions that were bound by TRIM28, and gained accessibility in the T28KO hESCs. Using the WGBS dataset, we show that the average DNA methylation levels of the 477 differentially accessible regions were significantly lower in T28KO hESCs compared with Ctrls (Figure 4C). Using enrichment analysis, we discovered that these 477 regions were enriched in HERVs, LTRs, and LINE elements (Figure S4A and Table S3). Boxplot analysis shows that average DNA methylation levels were dramatically reduced in HERVs, LTRs, and LINEs in T28KO compared with Ctrl hESCs (Figures 4D–4F). Further analysis of the hypomethylated HERVs (which are mainly composed of HERVKs) showed that their expression levels are unaffected between Ctrl and T28KO primed hESCs (Figure S4B).

In addition to the 2,224 new ATAC-seq peaks in T28KO hESCs, we also discovered a large number of peaks (1,849) (Table S3) that changed from accessible in Ctrls to inaccessible in the T28KO hESCs. None of these peaks overlapped with TRIM28 binding by ChIP-seq (Theunissen et al., 2016). Loss of accessibility was particularly enriched in promoters, exons, 5′ UTRs, and coding regions (Figure S4C). To determine how decreased accessibility in gene promoters corresponded to changes in DNA methylation, we calculated the average DNA methylation in the accessible promoters in Ctrl hESCs and the corresponding DNA methylation levels of these same inaccessible promoters in T28KO hESCs (n = 121 promoters). We discovered that in Ctrl cells these promoters began with low levels of DNA methylation, whereas in T28KO hESCs the promoters switch from low to high levels of DNA methylation (Figures S4D and S4E). Similarly, promoters that switch from inaccessible to accessible in T28KO hESCs switch from high to low levels of DNA methylation (Figures S4D and S4E), indicating that the chromatin accessibility in promoters is clearly inversely correlated with DNA methylation.

TRIM28 Is Required to Maintain DNA Methylation at Paternal but Not Maternal ICRs in Primed hESCs

Given previous reports that TRIM28 is required to maintain DNA methylation at IAPs in mouse embryos and also at ICRs (Alexander et al., 2015; Messerschmidt et al., 2012; Quenneville et al., 2011), we next evaluated the role of TRIM28 at ICRs in primed hESCs. Notably, TRIM28 is not enriched at any of the maternal ICRs in primed hESCs (n = 29) and instead is only enriched at the two paternal ICRs called *IG-DMR* (Figure 5A) and the *H19* ICR (Figure 5B). This result is different from that in the mouse where TRIM28 is enriched at both maternal and paternal ICRs in mouse embryos (Alexander et al., 2015; Messerschmidt et al., 2012).

Next we evaluated DNA methylation levels at the maternal and paternal ICRs in Ctrl and T28KO hESCs. We discovered variable imprinting methylation levels at individual ICRs in hESCs as previously reported (International Stem Cell Initiative et al., 2007; Pastor et al., 2016; Rugg-Gunn et al., 2007). However, on average, DNA methylation levels at maternal ICRs in Ctrl and T28KO hESCs were around 50% (Figure 5C). At the *MEG3* paternally methylated imprinted locus, we discovered that the *IG-DMR* and the *MEG3* promoter were demethylated in the T28KOs, relative to Ctrls, and this was associated with increased chromatin accessibility (Figure 5D). A similar change in chromatin was also observed at the *H19* ICR (Figure 5D). Consistent with demethylation and the emergence of accessible chromatin, the expression levels of *MEG3* and *H19* RNA transcripts were both increased in T28KO cells relative to Ctrls (Figures 5E and 5F). Taken together, our results suggest that TRIM28 is required to continually target the paternal ICRs for DNA methylation. Critically, maternal ICRs in hESCs do not require TRIM28 to remain methylated.

To determine whether the effects at the *MEG3* promoter were direct, we evaluated TRIM28 binding by chromatin immunoprecipitation followed by PCR (ChIP-qPCR) (Figure 5G). Our results show that TRIM28 is bound at the *MEG3* promoter in Ctrl cells, with no binding in the T28KOs. Therefore TRIM28 is most likely acting on both the *IG-DMR* and the *MEG3* promoter. Similarly, we also confirmed that TRIM28 is bound at the *H19* ICR in Ctrl hESCs (Figure 5H), and this binding is lost in the T28KOs. We also performed ChIP-qPCR for histone h3 lysine 9 trimethylation (H3K9me3), which is a known effector of TRIM28 binding on account of TRIM28's interaction with SETDB1 (Schultz et al., 2002). Our data show

(E and F) Relative expression using real-time RT-PCR of *MEG3* and *H19* in T28KO(U1-9) primed hESCs relative to Ctrl. ****$p < 0.0001$. Error bars represent SD.
(G–J) ChIP-qPCR of TRIM28 and H3K9me3 at the MEG3 promoter and H19-ICR, in Ctrl and T28KO(U1-9) hESCs.
(K) Sanger sequencing of SNPs in primed Ctrl and T28KO(U1-9) hESCs and naive hESCs as a control for biallelic expression. Arrows indicate two independent H19 SNPs.

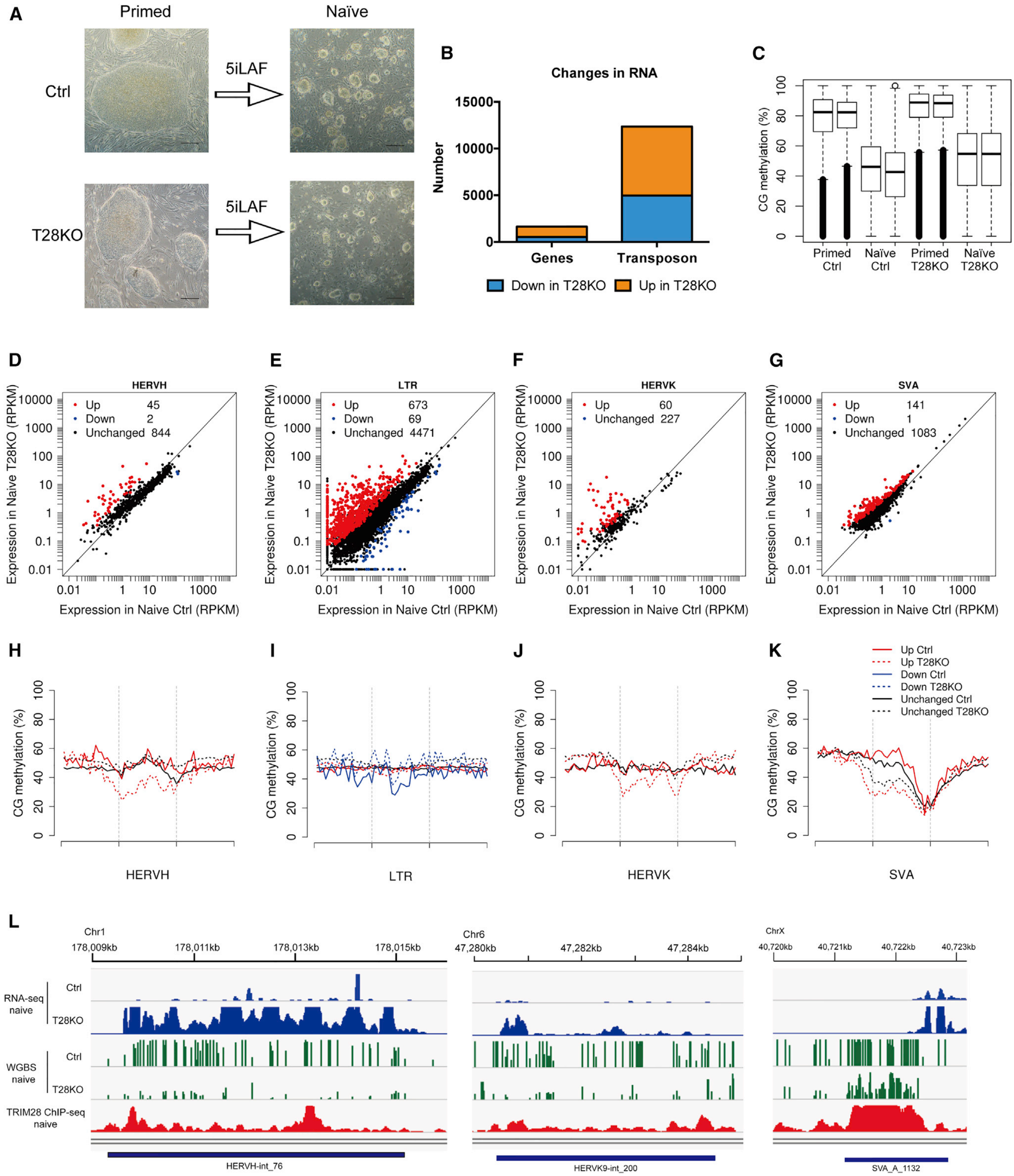

Figure 6. Naive hESCs Are Dependent upon TRIM28 for TE Repression but Not Self-Renewal

(A) Morphology of 5iLAF reverted Ctrl and T28KO(U1-9) naive hESCs. Scale bars, 100 μm.

(B) RNA-seq of Ctrl and T28KO(U1-9) naive hESCs showing DEGs (n = 1,638) and DETEs (n = 12,350).

(legend continued on next page)

that H3K9me3 is also reduced at the *MEG3* promoter, as well as the *H19* ICR in T28KO cells (Figures 5I and 5J). To determine whether this loss of methylation affects allelic expression, we examined the SNP in the *H19* RNA transcribed in the UCLA1 hESC line (Pastor et al., 2016). Using these SNPs as a reference, we show that TRIM28 is required to prevent loss of imprinting and maintain monoallelic expression of *H19* in self-renewing hESCs (Figure 5K). Taken together, TRIM28 plays highly localized and precise roles in the paternally methylated ICRs where it acts to promote H3K9me3 and DNA methylation in the self-renewing primed state.

TRIM28 Represses a Broad Range of Transposable Elements in Naive hESCs

Major transcriptional and epigenetic changes occur when primed hESCs are reverted to the naive state. Most notably, ICRs lose DNA methylation, LTR7-HERVH TEs are repressed, and SVA TEs are induced (Pastor et al., 2016; Theunissen et al., 2016). To evaluate the effects of TRIM28 upon reversion to the naive state, we reverted Ctrl and T28KO hESCs in the ground-state naive medium called 5iLAF (Theunissen et al., 2014). Starting from passages 3–5 post reversion, both Ctrl and T28KO putative naive hESC exhibited typical small, round colonies (Figure 6A). We have successfully generated naive hESC lines from both T28KO(U1-9) and T28KO(U1-11) primed hESCs (data not shown). RNA-seq of stage-specific embryonic antigen 4 (SSEA4)-negative, TRA-1–85-positive naive hESCs (Pastor et al., 2016) from Ctrl and T28KO 5iLAF cultures revealed that primed-specific genes *OTX2*, *ZIC2*, *ZIC3*, and *ZIC5* were downregulated in naive T28KO and Ctrl hESCs, whereas the naive-specific genes *KLFs*, *DPPA3*, and *TFCP2L1* were significantly upregulated. The RNA-seq analysis did reveal that T28KO naive hESCs had lower average expression of *NANOG* (fold change = 1.82) and *DPPA3* (fold change = 1.80) compared with Ctrl hESCs (Figure S5A), although this did not reach statistical significance (defined as fold change $\geq$ 2, false discovery rate < 0.05). No morphological differences in colonies were observed between Ctrl and T28KO naive hESCs under phase-contrast microscopy, suggesting that a null mutation in TRIM28 is also compatible with naive stem cell self-renewal in human. However, we did discover that T28KO naive hESCs grow more slowly than Ctrl naive hESCs (Figure S5B). We used array comparative genomic hybridization (aCGH) to determine copy number variants (CNVs) in these two cell lines, which showed that the naive Ctrl hESCs have normal CNVs while the T28KO naive hESCs have two deletions at chromosome 20 and chromosome 22 that do not overlap with normal CNVs (Figure S5C).

Although TE expression is altered in primed hESCs with a null mutation in *TRIM28*, ChIP-seq data of TRIM28 in wild-type 5iLAF cultures shows that significantly more TEs are bound by TRIM28 under 5iLAF naive conditions (Figure S5D). Consistent with this, reverting *TRIM28* null hESCs to the naive state results in 12,350 DETEs (Figure 6B and Table S2). Enrichment analysis of the DETE identified HERV and LTR families, as well as SVAs (Figure S5E). Similar to the effects of TRIM28 on primed hESCs, we also discovered 1,638 DEGs (Figure S5F).

We next evaluated changes in DNA methylation in naive T28KO cells relative to Ctrl cells (Figure 6C). As expected, reversion to the naive state also resulted in a global loss of DNA methylation from an average of 74.5% in primed cells to an average of 42.4% in the naive state. In naive T28KO hESCs we discovered that DNA methylation was also globally reduced (Figure 6C). However, the average levels of global DNA methylation were slightly higher in the naive T28KOs (50.15% versus 42.4%). This translated to significantly more hypermethylated DMRs in the T28KO hESCs (2,552) and only a small number of hypomethylated regions (578). Notably, the hypomethylated DMRs were particularly enriched in SVAs and HERVs (Figure S5G and Table S3).

To determine the relationship between TRIM28 and DNA methylation in the hypomethylated naive state, we combined the ChIP-seq dataset of TRIM28 in naive hESCs (Theunissen et al., 2016), with the RNA-seq and WGBS data of T28KO and Ctrl naive hESCs generated in this study. This comparison identified subfamilies of TRIM28 bound HERVHs, HERVKs, and SVAs that were significantly affected at both the RNA and DNA methylation level (Figures 6D–6K and Table S4). Notably, HERVH family members were both up- and downregulated in T28KO hESCs (Figure 6D), with upregulated HERVHs exhibiting an extra peak of TRIM28 binding in wild-type cells (Figures S5H and 6L). Of the 60 HERVH elements that were identified as upregulated in primed hESCs, 15 of 60 remained

(C) Box plots showing percentage of CpG methylation levels in Ctrl and T28KO(U1-9) hESCs in the primed and naive states (n = 2 independent samples of each genotype in each pluripotent state).
(D–G) Scatterplots showing selected DETEs between Ctrl and T28KO(U1-9) naive hESCs. Red dots, significantly upregulated; blue dots, significantly downregulated; black dots, unchanged. Expression values are represented as RPKM.
(H–K) Percent CpG methylation of TEs shown in (D) to (G). Dotted lines, CpG methylation in T28KO; solid lines, the same regions in Ctrl.
(L) Representative tracks of RNA-seq, WGBS, and ChIP-seq at a representative HERVH, HERVK, and SVA.
See also Figure S5.

upregulated following reversion to the naive state. However, 60 HERVH family members were newly upregulated in naive T28KO hESCs relative to Ctrl, including the primed-specific LTR7-HERVHs (Table S4). Similarly LTRs were also highly enriched in TRIM28 binding in the naive state, and consistent with this, T28KO naive cells had increased RNA expression from LTRs (Figure 6E). Interestingly, the differentially upregulated LTRs did not show obvious loss of DNA methylation below the genome average, indicating that LTRs are regulated primarily by TRIM28 (Figure 6I). In the primed state, TRIM28 null mutations had no significant effect on HERVK RNA expression (Figure 3D). In contrast, HERVK elements were now derepressed in T28KO naive hESCs relative to Ctrls (Figure 6F), and this was also accompanied by changes in DNA methylation (Figures 6J and 6L). Therefore, expression of HERVK subfamily members was particularly sensitive to loss of TRIM28 in the naive state compared with the primed state.

Although naive human pluripotency under wild-type conditions is associated with expression of SVAs at the expense of LTR7-HERVH, a TRIM28 null mutation in naive hESCs leads to further upregulation of SVA family members, and this is associated with loss of methylation particularly at their 5′ regions (Figures 6G and 6K). Taken together, TRIM28 has an important role in regulating the expression and DNA methylation levels of TEs in the naive state, particularly the repression of LTR7-HERVHs, HERVKs and SVAs.

DISCUSSION

In the current study, we show that self-renewal of hESCs in the naive and primed states of human pluripotency are compatible with significant changes in TE expression, accompanied by changes in chromatin accessibility and DNA methylation, particularly at HERVs and LTRs. This result is unlike the biological response in mouse pluripotent cells, where a *Trim28* null mutation in mouse cells is incompatible with mouse ESC self-renewal and mouse embryo development (Cammas et al., 2000; Rowe et al., 2010). One explanation for these species-specific differences in response to loss of TRIM28 is that the primed state of hESC pluripotency is more similar to mouse epiblast stem cells (EpiSCs) than mouse ESCs (Tesar et al., 2007). Generating a TRIM28 null mutation in mouse EpiSCs in future studies will address this issue.

It is also possible that the modest biological effect on human pluripotent stem cell self-renewal in the absence of human TRIM28 relative to the mouse is a consequence of the unique repertoire of provirus that infected and expanded in rodent genomes relative to humans, where it is possible that the rodent TEs today are more mutagenic and/or deleterious when derepressed in the absence of TRIM28 (Dewannieux et al., 2004; Zhang et al., 2008). In the human genome, most TEs are ancient non-functional relics of past proviral infections that have lost transposition competency. Furthermore, our data also suggest that the continued targeting of TRIM28 to TE-associated chromatin is not the primary defense for most TE repression in human pluripotent stem cells, because a large fraction of the TRIM28-bound TEs remained unperturbed in both the primed and the naive state when TRIM28 was deleted.

Given that hESCs with a TRIM28 null mutation are biased against hPGCLC differentiation, we speculate that this occurs as an unintended consequence of dynamically changing accessibility of local chromatin in human primed pluripotent hESCs. The differentiation of hPGCLCs from pluripotent stem cells requires a narrow window in development where the same signaling pathways used to generate primitive streak (notably, ACTIVIN, WNTs, and bone morphogenetic protein 4 [BMP4]) are also required to specify hPGCLCs. It was previously reported that changes to the concentrations of growth factors that promote hPGCLC differentiation also significantly affect germline competency (Sasaki et al., 2015). We propose that for lineages where the window for competency is very narrow (such as the allocation of hPGCLCs from hESCs), small changes in the response to somatic cell signaling cues bias against germline fate.

We also discovered that unlike mouse ESCs where mouse TRIM28 regulates DNA methylation at almost all ICRs (Alexander et al., 2015), human TRIM28 is only responsible for regulating DNA methylation at the paternally methylated ICRs in human pluripotent stem cells. Previous studies have shown that ICR methylation in primed hESCs is not regulated by the *de novo* DNA methyltransferases DNMT3A or DNMT3B (Liao et al., 2015). These results may suggest that DNMT1 re-targets discrete paternal ICRs and promoters together with TRIM28 outside of replication-coupled DNA methylation maintenance, given that the paternally methylated ICRs were so specifically and discretely targeted. Alternatively, it is conceivable that a previously undiscovered DNMT may have evolved to interact with TRIM28 to target DNA methylation specifically to paternal ICRs.

TRIM28 itself has no sequence specificity. Instead, TRIM28 binds to ZNFs, which target TRIM28 to discrete sites in a sequence-dependent manner. The ZNF responsible for targeting TRIM28 to ICRs in the mouse genome (ZFP57) is also responsible for some ICR methylation in the human genome. Specifically, a homozygous deletion of ZFP57 in human is compatible with life, germ cell development, and fertility, with homozygous children exhibiting variable ICR hypomethylation mostly at maternal ICRs (Mackay et al., 2008). In light of our findings it is possible that ZFP57 targets maternal ICRs for DNA

methylation in a TRIM28-independent mechanism, or alternatively that TRIM28 targets paternal ICRs by a ZFP57-independent mechanism. Future studies could be designed to address these hypotheses.

Previous reports have shown that in the majority of reversions in 5iLAF, hESCs exhibit karyotypic instability (Pastor et al., 2016; Theunissen et al., 2014). In the current study, the Ctrl naive hESCs had a normal karyotype based on CNV analysis. However, we discovered that the T28KO naive hESCs cultured under identical conditions developed abnormal CNVs. It has previously been reported that SVA expression causes genome instability and even diseases (Kaer and Speek, 2013). Therefore, we hypothesize that one of the possible mechanisms associated with increased genome instability may be due to increased SVA expression in the naive state.

Taken together, TRIM28 has broadly conserved roles in human and mouse pluripotent stem cells where it functions to regulate TE expression, and this is associated with changes in the landscape of chromatin accessibility and DNA methylation. However, our data also highlight species-specific differences in the naive state of pluripotency including the finding that human naive pluripotent stem cells tolerate significant changes in TE expression downstream of a TRIM28 mutation. We also show, surprisingly, that TRIM28 has a preference for targeting paternal ICRs in the primed self-renewing state rather than ICRs more broadly. We were also able to uncover a role for TRIM28 in germline competency, and future studies will involve identifying the ZNFs responsible for these different phenotypes.

EXPERIMENTAL PROCEDURES

Primed and Naive hESC Culture

Primed and naive hESCs were cultured as previously described (Pastor et al., 2016). See Supplemental Experimental Procedures for details. All hESC studies were approved by the UCLA Embryonic Stem Cell Research Oversight (ESCRO) Committee.

Generation of TRIM28 Knockout Primed hESC Line

Taking advantage of the CRISPR/Cas9 targeting technology in mammalian cells (Cong et al., 2013), we designed paired gRNAs that target exon 4 and exon 11 of the human *TRIM28* genome, respectively, using the crispr.mit.edu web site, and ligated the gRNAs with px459 vector to obtain the final constructs. Two micrograms of each targeting vector (4 μg in total) were electroporated into 800K human ESCs, and we used the P3 Primary Cell 4D-Nucleofector X Kit to perform the nucleofection following manufacturer's instructions. The cells were transferred to one well of a 24-well plate with feeders post nucleofection, and on the next day, 0.35 μg/mL puromycin was added to the medium as a primary screen of successfully electroporated cells for 24 hr. The medium was then changed back to regular hESC medium + ROCKi for another 3 days until cells were densely grown. Cells were then split by Accutase and transferred as 2K and 10K per dish onto 10-cm dishes with feeders. After 10–14 days when the colonies were big enough, single colonies were picked manually, and the genomic DNA of each line was extracted and genotyped. The homozygous *TRIM28* knockout lines were then expanded for further analysis. The gRNA sequences are: 5′-ACG TTC ACC ATC CCG AGA CT-3′ for exon 4 and 5′-GGT GAG CGG CCT TAT GCG CA-3′ for exon 11.

hPGCLC Differentiation

Primed hESCs were differentiated into hPGCLC as described in Sasaki et al. (2015) with some modifications. Day-7 hESCs were dissociated into single cells with 0.05% trypsin-EDTA (Gibco) and plated onto a human plasma fibronectin (Invitrogen)-coated 12-well-plate at 200,000 cells/well cell density in 2 mL/well of incipient mesoderm-like cells (iMeLCs) medium, which is composed of 15% knockout serum replacement (KSR), 1× non-essential amino acids (NEAA), 0.1 mM 2-mercaptoethanol, 1× penicillin-streptomycin-glutamine (Gibco), 1 mM sodium pyruvate (Gibco), 50 ng/mL Activin A (Peprotech), 3 μM CHIR99021 (Stemgent), 10 μM ROCKi (Y27632, Stemgent), and 50 ng/mL primocin in Glasgow's minimal essential medium (GMEM) (Gibco). Twenty-four hours later, iMeLCs were dissociated into single cells by 0.05% trypsin-EDTA, followed by plating onto ultra-low cell attachment U-bottom 96-well plates (Corning) at a density of 3,000 cells/well in 200 μL/well of hPGCLC medium, which is composed of 15% KSR, 1× NEAA, 0.1 mM 2-mercaptoethanol, 1× penicillin-streptomycin-glutamine (Gibco), 1 mM sodium pyruvate (Gibco), 10 ng/mL human leukemia inhibitory factor (Millipore), 200 ng/mL human BMP4 (R&D systems), 50 ng/mL human epidermal growth factor (R&D Systems), 10 μM of ROCKi (Y27632, Stemgent), and 50 ng/mL primocin in GMEM (Gibco). Day-4 hPGCLC aggregates were used for further analysis.

hPGCLC Flow-Cytometry Analysis

Day-4 hPGCLC aggregates were dissociated with 0.05% trypsin-EDTA for 10 min at 37°C. The dissociated cells were stained with antibodies, which were INTEGRINα6 conjugated with BV421 (BioLegend) and EpCAM conjugated with 488 (BioLegend) for at least 1 hr on ice. Cells were then washed with FACS buffer (1% BSA in PBS) once and resuspended in FACS buffer with 7-aminoactinomycin D (BD Pharmingen). Finally, cells were passed through a 40-μm cell strainer (Fisher Scientific) and used for flow-cytometric analysis.

RNA-Seq

Cells were centrifuged and cell pellets lysed in 350 μL of RLT buffer, and total RNA was extracted using an RNeasy micro kit (Qiagen) or RNeasy mini kit (Qiagen). cDNA was amplified using Ovation RNA-Seq System V2 (Nugen) according to the manufacturer's instructions. Amplified cDNA was then sheared to ~200 bp length by a Covaris S220 Focused ultrasonicator. RNA-seq libraries were constructed by using Ovation Rapid Library Systems (Nugen, 0319-32 for index 1–8 and 0320-32 for index 9–16) and quantified by a KAPA library quantification kit (Illumina). Libraries were

subjected to single-end 50-bp sequencing on a HiSeq 2500/4000 sequencer with 4–6 indexed libraries per lane.

ATAC-Seq

Cells were lysed directly in lysis buffer (500 μL 1 M Tris [pH 7.4], 100 μL 5 M NaCl, 150 μL 1 M $MgCl_2$, 500 μL 10% NP-40 in 50 mL water). After centrifugation, the cell pellet was resuspended in transposase buffer, which contained the Tn5 transposase enzyme and tagmentation buffer (Nextera DNA library prep kit: Illumina, catalog no. 15028212), and incubated at 37°C for 30 min. After purification by a MinElute PCR Purification Kit 250 (Qiagen 28006), P1 barcode (25 μM) and appropriate P2 barcodes (25 μM) were added to the DNA and run for five cycles of PCR reaction (NEBNext High-Fidelity 2× PCR Master Mix; NEB, M0541S). Ten percent of the PCR product was taken out for real-time PCR analysis to determine the amplification cycles of the library, followed immediately by PCR amplification of the remaining library DNA. Libraries were subjected to paired-end 50-bp sequencing on a Hi Seq 4000 sequencer with 4–6 indexed libraries per lane.

Whole-Genome Bisulfite Sequencing

DNA for bisulfite sequencing was extracted using the Quick gDNA Mini-Prep Kit (Zymo D3025) and quantified using the Qubit dsDNA High Sensitivity Kit (Life Technologies). Bisulfite sequencing libraries were prepared using the Ovation Ultralow Methyl-Seq Library System (Nugen, 0335 for DR Multiplex System 1–8, 0336 for DR Multiplex System 9–16). Libraries were subjected to single-end 100-bp sequencing on HiSeq 4000 sequencer with about 1 sample per lane to achieve coverage.

ACCESSION NUMBERS

The accession number for the RNA-seq, ATAC-seq, and WGBS data reported in this paper is GEO: GSE99215.

SUPPLEMENTAL INFORMATION

Supplemental Information includes Supplemental Experimental Procedures, five figures, and four tables and can be found with this article online at https://doi.org/10.1016/j.stemcr.2017.11.020.

AUTHOR CONTRIBUTIONS

A.T.C. conceived and supervised the project and wrote the manuscript. Y.T. performed experiments and data analyses and wrote the manuscript. P-.Y.C. and M-.R.Y. performed bioinformatics analyses and helped write the manuscript. T.C. performed ChIP-qPCR and RT-qPCR and helped with CM differentiation. H.N. and A.N. performed the CM differentiation assay. R.K. and L.H. performed the teratoma assay. Y.C.T. performed experiments and provided technical assistance.

ACKNOWLEDGMENTS

We thank the UCLA Broad Stem Cell Research Center (BSCRC) Flow Cytometry core and the UCLA BSCRC High Throughput Sequencing Core for their assistance. This work is supported by a grant from the NIH, R01 HD079546 to A.T.C.; funds from the UCLA Broad Stem Cell Research Center and NIH NCATS UCLA CTSI, grant number UL1TR0001881; and by grants from Academia Sinica and National Health Research Institutes, Taiwan (NHRI- EXI03-10324SC) to P.-Y.C.

Received: June 25, 2017
Revised: November 27, 2017
Accepted: November 28, 2017
Published: December 28, 2017

REFERENCES

Alexander, K.A., Wang, X., Shibata, M., Clark, A.G., and García-García, M.J. (2015). TRIM28 controls genomic imprinting through distinct mechanisms during and after early genome-wide reprogramming. Cell Rep. *13*, 1194–1205.

Cammas, F., Mark, M., Dollé, P., Dierich, A., Chambon, P., and Losson, R. (2000). Mice lacking the transcriptional corepressor TIF1beta are defective in early postimplantation development. Development *127*, 2955–2963.

Cong, L., Ran, F.A., Cox, D., Lin, S., Barretto, R., Habib, N., Hsu, P.D., Wu, X., Jiang, W., Marraffini, L.A., et al. (2013). Multiplex genome engineering using CRISPR/Cas systems. Science *339*, 819–823.

Cordaux, R., and Batzer, M.A. (2009). The impact of retrotransposons on human genome evolution. Nat. Rev. Genet. *10*, 691–703.

Dewannieux, M., Dupressoir, A., Harper, F., Pierron, G., and Heidmann, T. (2004). Identification of autonomous IAP LTR retrotransposons mobile in mammalian cells. Nat. Genet. *36*, 534–539.

Diaz Perez, S.V., Kim, R., Li, Z., Marquez, V.E., Patel, S., Plath, K., and Clark, A.T. (2012). Derivation of new human embryonic stem cell lines reveals rapid epigenetic progression in vitro that can be prevented by chemical modification of chromatin. Hum. Mol. Genet. *21*, 751–764.

Fadloun, A., Le Gras, S., Jost, B., Ziegler-Birling, C., Takahashi, H., Gorab, E., Carninci, P., and Torres-Padilla, M.E. (2013). Chromatin signatures and retrotransposon profiling in mouse embryos reveal regulation of LINE-1 by RNA. Nat. Struct. Mol. Biol. *20*, 332–338.

Göke, J., Lu, X., Chan, Y.S., Ng, H.H., Ly, L.H., Sachs, F., and Szczerbinska, I. (2015). Dynamic transcription of distinct classes of endogenous retroviral elements marks specific populations of early human embryonic cells. Cell Stem Cell *16*, 135–141.

Grow, E.J., Flynn, R.A., Chavez, S.L., Bayless, N.L., Wossidlo, M., Wesche, D.J., Martin, L., Ware, C.B., Blish, C.A., Chang, H.Y., et al. (2015). Intrinsic retroviral reactivation in human preimplantation embryos and pluripotent cells. Nature *522*, 221–225.

Herbst, H., Sauter, M., and Mueller-Lantzsch, N. (1996). Expression of human endogenous retrovirus K elements in germ cell and trophoblastic tumors. Am. J. Pathol. *149*, 1727–1735.

Huntley, S., Baggott, D.M., Hamilton, A.T., Tran-Gyamfi, M., Yang, S., Kim, J., Gordon, L., Branscomb, E., and Stubbs, L. (2006). A comprehensive catalog of human KRAB-associated zinc finger genes: insights into the evolutionary history of a large family of transcriptional repressors. Genome Res. *16*, 669–677.

International Stem Cell Initiative, Adewumi, O., Aflatoonian, B., Ahrlund-Richter, L., Amit, M., Andrews, P.W., Beighton, G., Bello,

P.A., Benvenisty, N., Berry, L.S., et al. (2007). Characterization of human embryonic stem cell lines by the International Stem Cell Initiative. Nat. Biotechnol. *25*, 803–816.

Jachowicz, J.W., Bing, X., Pontabry, J., Bošković, A., Rando, O.J., and Torres-Padilla, M.E. (2017). LINE-1 activation after fertilization regulates global chromatin accessibility in the early mouse embryo. Nat. Genet. *49*, 1502–1510.

Kaer, K., and Speek, M. (2013). Retroelements in human disease. Gene *518*, 231–241.

Liao, J., Karnik, R., Gu, H., Ziller, M.J., Clement, K., Tsankov, A.M., Akopian, V., Gifford, C.A., Donaghey, J., Galonska, C., et al. (2015). Targeted disruption of DNMT1, DNMT3A and DNMT3B in human embryonic stem cells. Nat. Genet. *47*, 469–478.

Lu, X., Sachs, F., Ramsay, L., Jacques, P.É., Göke, J., Bourque, G., and Ng, H.H. (2014). The retrovirus HERVH is a long noncoding RNA required for human embryonic stem cell identity. Nat. Struct. Mol. Biol. *21*, 423–425.

Mackay, D.J.G., Callaway, J.L.A., Marks, S.M., White, H.E., Acerini, C.L., Boonen, S.E., Dayanikli, P., Firth, H.V., Goodship, J.A., Haemers, A.P., et al. (2008). Hypomethylation of multiple imprinted loci in individuals with transient neonatal diabetes is associated with mutations in ZFP57. Nat. Genet. *40*, 949–951.

Messerschmidt, D.M., de Vries, W., Ito, M., Solter, D., Ferguson-Smith, A., and Knowles, B.B. (2012). Trim28 is required for epigenetic stability during mouse oocyte to embryo transition. Science *335*, 1499–1502.

Pastor, W.A., Chen, D., Liu, W., Kim, R., Sahakyan, A., Lukianchikov, A., Plath, K., Jacobsen, S.E., and Clark, A.T. (2016). Naive human pluripotent cells feature a methylation landscape devoid of blastocyst or germline memory. Cell Stem Cell *18*, 323–329.

Quenneville, S., Verde, G., Corsinotti, A., Kapopoulou, A., Jakobsson, J., Offner, S., Baglivo, I., Pedone, P.V., Grimaldi, G., Riccio, A., et al. (2011). In Embryonic stem cells, ZFP57/KAP1 recognize a methylated hexanucleotide to affect chromatin and DNA methylation of imprinting control regions. Mol. Cell *44*, 361–372.

Quenneville, S., Turelli, P., Bojkowska, K., Raclot, C., Offner, S., Kapopoulou, A., and Trono, D. (2012). The KRAB-ZFP/KAP1 system contributes to the early embryonic establishment of site-specific DNA methylation patterns maintained during development. Cell Rep. *2*, 766–773.

Rotman, G., Itin, A., and Keshet, E. (1986). Promoter and enhancer activities of long terminal repeats associated with cellular retrovirus-like (VL30) elements. Nucleic Acids Res. *14*, 645–658.

Rowe, H.M., Jakobsson, J., Mesnard, D., Rougemont, J., Reynard, S., Aktas, T., Maillard, P.V., Layard-Liesching, H., Verp, S., Marquis, J., et al. (2010). KAP1 controls endogenous retroviruses in embryonic stem cells. Nature *463*, 237–240.

Rugg-Gunn, P.J., Ferguson-Smith, A.C., and Pedersen, R.A. (2007). Status of genomic imprinting in human embryonic stem cells as revealed by a large cohort of independently derived and maintained lines. Hum. Mol. Genet. *16*, R243–R251.

Sasaki, K., Yokobayashi, S., Nakamura, T., Okamoto, I., Yabuta, Y., Kurimoto, K., Ohta, H., Moritoki, Y., Iwatani, C., Tsuchiya, H., et al. (2015). Robust in vitro induction of human germ cell fate from pluripotent stem cells. Cell Stem Cell *17*, 178–194.

Schultz, D.C., Friedman, J.R., and Rauscher, F.J. (2001). Targeting histone deacetylase complexes via KRAB-zinc finger proteins: the PHD and bromodomains of KAP-1 form a cooperative unit that recruits a novel isoform of the Mi-2α subunit of NuRD. Genes Dev. *15*, 428–443.

Schultz, D.C., Ayyanathan, K., Negorev, D., Maul, G.G., and Rauscher, F.J. (2002). SETDB1: a novel KAP-1-associated histone H3, lysine 9-specific methyltransferase that contributes to HP1-mediated silencing of euchromatic genes by KRAB zinc-finger proteins. Genes Dev. *16*, 919–932.

Svoboda, P., Stein, P., Anger, M., Bernstein, E., Hannon, G.J., and Schultz, R.M. (2004). RNAi and expression of retrotransposons MuERV-L and IAP in preimplantation mouse embryos. Dev. Biol. *269*, 276–285.

Tesar, P.J., Chenoweth, J.G., Brook, F.A., Davies, T.J., Evans, E.P., Mack, D.L., Gardner, R.L., and McKay, R.D.G. (2007). New cell lines from mouse epiblast share defining features with human embryonic stem cells. Nature *448*, 196–199.

Theunissen, T.W., Powell, B.E., Wang, H., Mitalipova, M., Faddah, D.A., Reddy, J., Fan, Z.P., Maetzel, D., Ganz, K., Shi, L., et al. (2014). Systematic identification of culture conditions for induction and maintenance of naive human pluripotency. Cell Stem Cell *15*, 471–487.

Theunissen, T.W., Friedli, M., He, Y., Planet, E., O'Neil, R.C., Markoulaki, S., Pontis, J., Wang, H., Iouranova, A., Imbeault, M., et al. (2016). Molecular criteria for defining the naive human pluripotent state. Cell Stem Cell *19*, 502–515.

Turelli, P., Castro-Diaz, N., Marzetta, F., Kapopoulou, A., Raclot, C., Duc, J., Tieng, V., Quenneville, S., and Trono, D. (2014). Interplay of TRIM28 and DNA methylation in controlling human endogenous retroelements. Genome Res. *24*, 1260–1270.

Valle-García, D., Qadeer, Z.A., McHugh, D.S., Ghiraldini, F.G., Chowdhury, A.H., Hasson, D., Dyer, M.A., Recillas-Targa, F., and Bernstein, E. (2016). ATRX binds to atypical chromatin domains at the 3′ exons of zinc finger genes to preserve H3K9me3 enrichment. Epigenetics *11*, 398–414.

Wang, J., Xie, G., Singh, M., Ghanbarian, A.T., Rasko, T., Szvetnik, A., Cai, H., Besser, D., Prigione, A., Fuchs, N.V., et al. (2014). Primate-specific endogenous retrovirus-driven transcription defines naive-like stem cells. Nature *516*, 405–409.

Wolf, D., and Goff, S.P. (2007). TRIM28 mediates primer binding site-targeted silencing of murine leukemia virus in embryonic cells. Cell *131*, 46–57.

Zhang, Y., Maksakova, I.A., Gagnier, L., van de Lagemaat, L.N., and Mager, D.L. (2008). Genome-Wide assessments reveal extremely high levels of polymorphism of two active families of mouse endogenous retroviral elements. PLoS Genet. *4*, e1000007.

Zuo, X., Sheng, J., Lau, H.-T., McDonald, C.M., Andrade, M., Cullen, D.E., Bell, F.T., Iacovino, M., Kyba, M., Xu, G., et al. (2012). Zinc finger protein ZFP57 requires its Co-factor to recruit DNA methyltransferases and maintains DNA methylation imprint in embryonic stem cells via its transcriptional repression domain. J. Biol. Chem. *287*, 2107–2118.

Stem Cell Reports, Volume *10*

Supplemental Information

TRIM28-Regulated Transposon Repression Is Required for Human Germline Competency and Not Primed or Naive Human Pluripotency

Yu Tao, Ming-Ren Yen, Tsotne Chitiashvili, Haruko Nakano, Rachel Kim, Linzi Hosohama, Yao Chang Tan, Atsushi Nakano, Pao-Yang Chen, and Amander T. Clark

Supplemental Figures

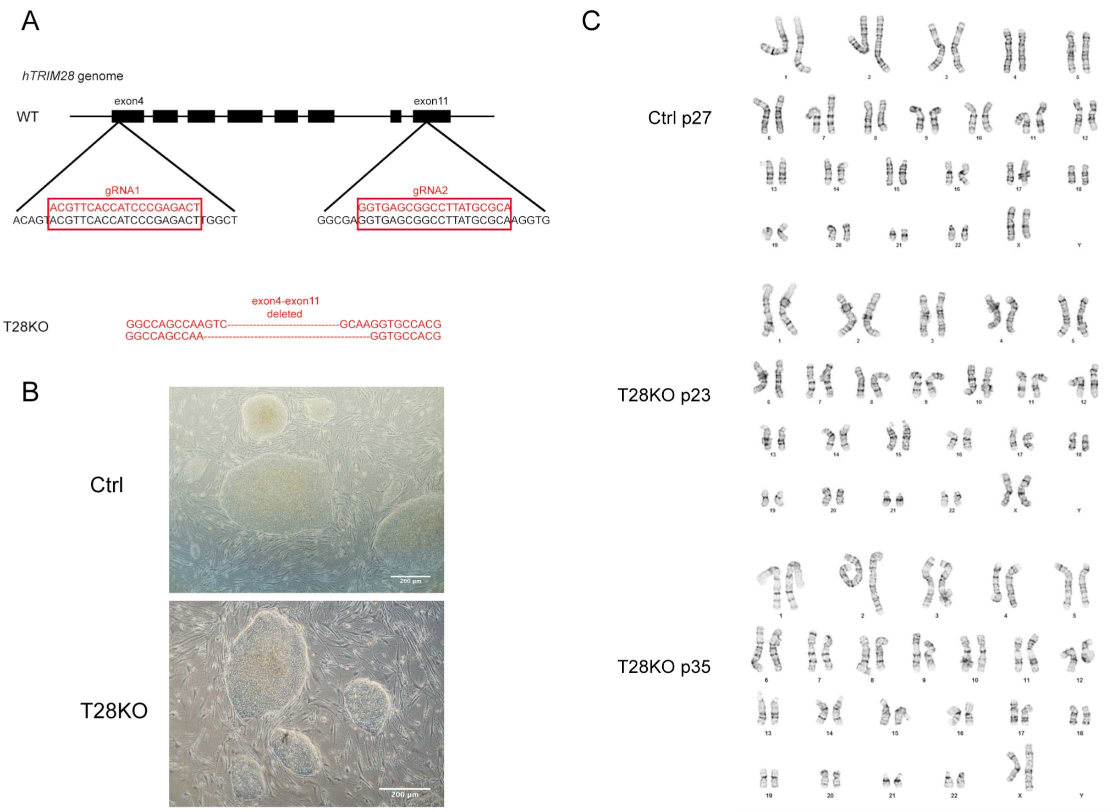

Figure S1 related to Figure 1
(A) Schematic overview of the TRIM28 locus and the CRISPR/Cas9 gRNA targeting sites. Guide RNA-targeting sequences are marked as red in the box. The TRIM28 mutation was confirmed by Sanger Sequencing DNA clones from the T28KO (U1-9) mutant cell line.
(B) Bright field images of primed Ctrl hESC and T28KO (U1-9) hESC, scale bar indicates 100µm.
(C) Karyotypes of Ctrl (passage number: 27) and T28KO (U1-9) (passage number: 23 and 35) primed hESCs.

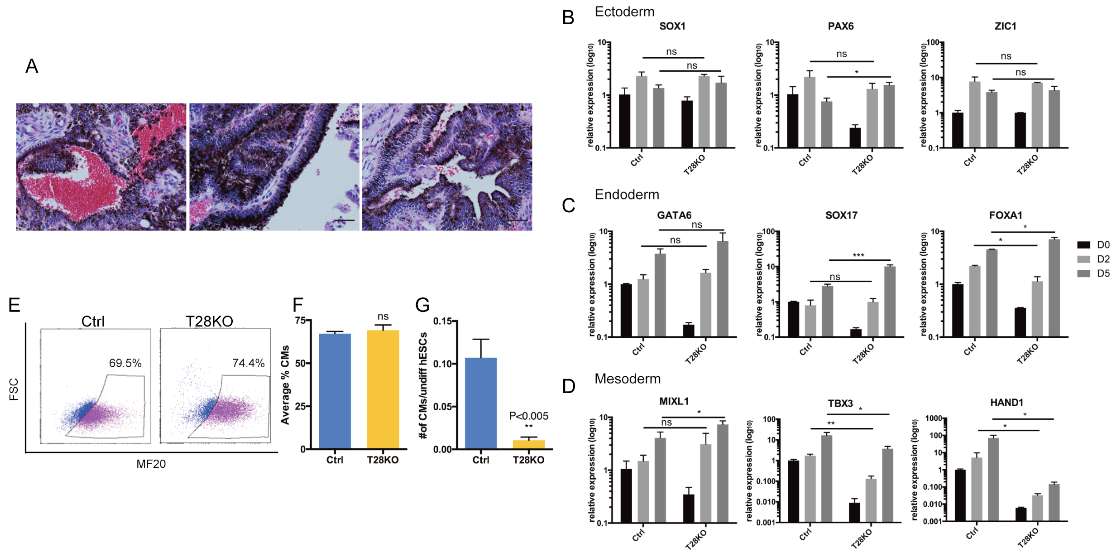

Figure S2 related to Figure 2

(A) Representative photos of teratomas from primed T28KO (U1-9) hESCs, detailed (hematoxylin and eosin) HE staining images show the highly pigmented cells in T28KO (U1-9) teratoma, scale bar = 50μm.

(B-D) RT-qPCR of representative genes of the 3 somatic germ layers, in undifferentiated hESC (D0), Day2, and Day5 of spontaneous EB differentiation from both Ctrl and T28KO (U1-9) hESCs, n=3 independent experiments, * indicates $p<0.05$, ** indicates $p<0.01$, ns= non significant, error bar = SD.

(E-G) Cardiomyocyte (CM) differentiation of Ctrl and T28KO (U1-9) hESC. (E) Representative flow cytometry results of Day14 EB, MF20 is a positive marker of CMs at this stage. (F) Average percentage of CM at Day14, n=3 independent experiments, $p>0.05$ by Student's t-test. (G) CM differentiation efficiency when the cell numbers of acquired CMs at Day 14 are normalized to the cell numbers of starting undifferentiated hESCs, error bar = SD.

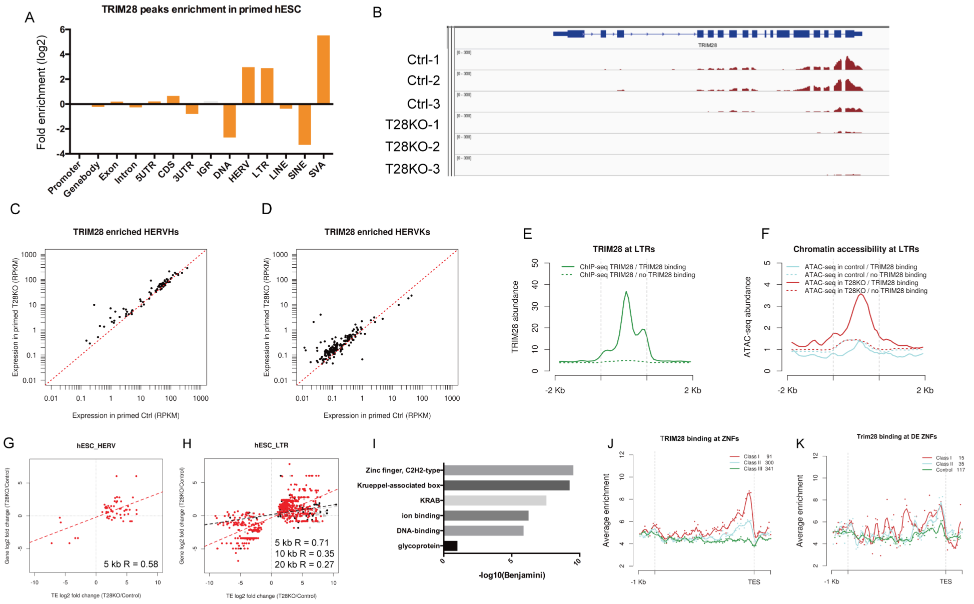

Figure S3 related to Figure 3
(A) Enrichment analysis of TRIM28 binding sites in wild type primed hESCs of ChIP-Seq data set from Theunissen et al.).
(B) Snapshots of RNA-seq peaks of Ctrl and T28KO (U1-9) primed hESCs at the TRIM28 locus, each with 3 replicates.
(C-D) Scatter plots of relative expression shown in Reads Per Kilobase of transcript per Million mapped reads (RPKM) of HERVH and HERVK in Ctrl and T28KO (U1-9) primed hESCs.
(E-F) Meta-plot of TRIM28 binding at LTRs (Meta analysis from the data set of Theunissen et al.) and chromatin accessibility at TRIM28 bound LTRs in Ctrl and T28KO (U1-9) hESCs.
(G-H) Correlation between differentially expressed HERVs / LTRs and differentially expressed neighboring genes.
(I) Gene ontology analysis of up-regulated genes in T28KO (U1-9) primed hESCs.
(J) TRIM28 binding at ZNFs in primed hESCs using the class I, class II and class III defined ZNFs identified by Valle-Garcia et al., in the human erythroleukemic cell line K562.
(K) Up-regulated ZNFs identified in T28KO (U1-9) hESCs correspond to class I and class II ZNFs, with Trim28 binding in the middle and 3’ end of the gene.

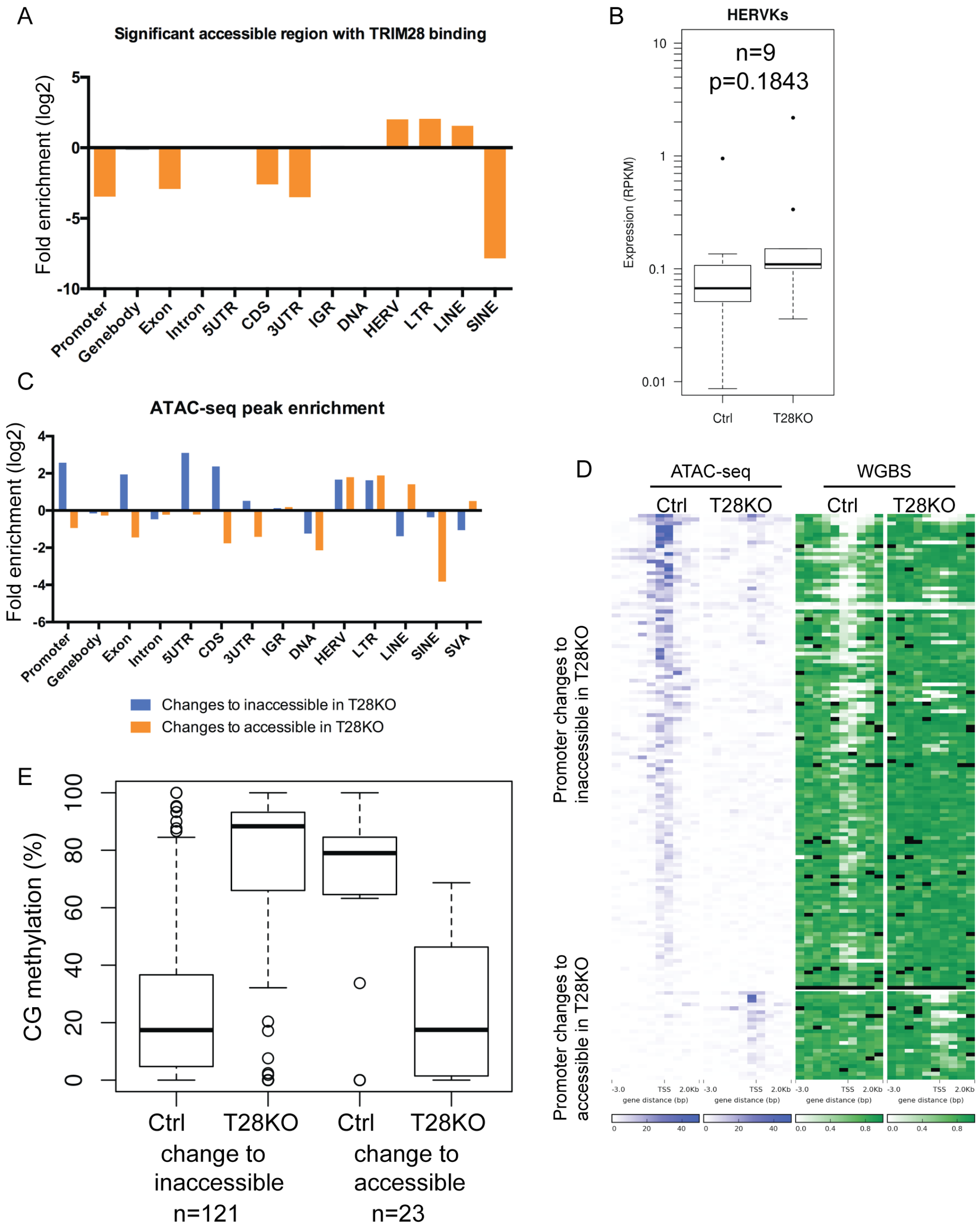

Figure S4 related to Figure 4

(A) Enrichment analysis of the regions bound by TRIM28 in wild type hESCs that become accessible in T28KO (U1-9) hESCs relative to Ctrl.

(B) Box-plot of the expression levels of the expressed HERVKs from Figure 4D.

(C) Enrichment analysis of the regions that changed from accessible to inaccessible in T28KO (U1-9) hESCs relative to Ctrl and from inaccessible to accessible in T28KO (U1-9) hESCs relative to Ctrl.

(D) Heatmap of promoters that change from accessible to inaccessible (n=121) and inaccessible to accessible (n=23) by ATAC-Seq in T28KO (U1-9) hESCs relative to Ctrls and the corresponding DNA methylation levels.

(E) Box-plot of percent CG DNA methylation of the same promoters from Figure 4D.

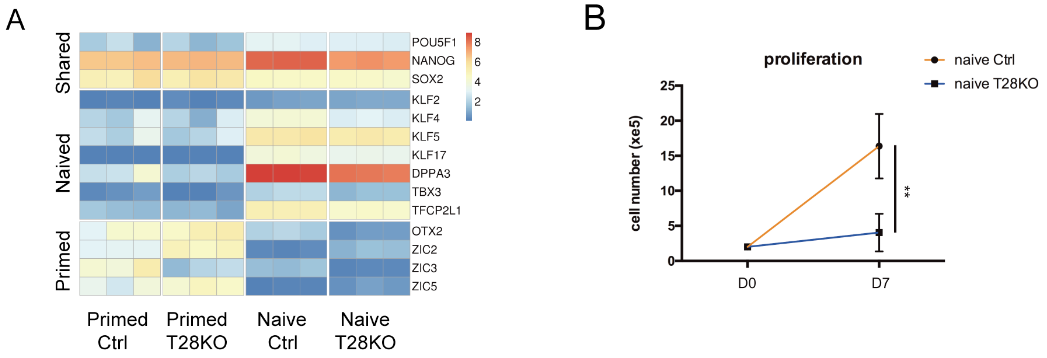

C

Ctrl

Amp/Deletion Table									
Chr	Amp/Del	Start (bp)	Stop (bp)	Size(kb)	Chr Band	# Probes	Log2 Ratio	Genes$^{\alpha}$	Overlap Normal CNVs?$^{\beta}$
6	Amp	168,371,416	168,581,212	210	q27	7	0.677991	MLLT4, HGC6.3, KIF25-AS1, KIF25, FRMD1	Yes
15	Amp	20,481,702	21,939,711	1,458	q11.1 - q11.2	5	0.522967	CHEK2P2, HERC2P3, GOLGA6L6, GOLGA8CP, NBEAP1, MIR3118-4, MIR3118-3, MIR3118-2, POTEB3, POTEB, POTEB2, NF1P2, MIR5701-3, MIR5701-1, MIR5701-2, LINC01193, LOC646214	Yes

Total Amp/Del: 2

T28KO

Amp/Deletion Table									
Chr	Amp/Del	Start (bp)	Stop (bp)	Size(kb)	Chr Band	# Probes	Log2 Ratio	Genes$^{\alpha}$	Overlap Normal CNVs?$^{\beta}$
6	Amp	168,371,416	168,581,212	210	q27	7	0.507282	MLLT4, HGC6.3, KIF25-AS1, KIF25, FRMD1	Yes
15	Amp	20,481,702	21,939,711	1,458	q11.1 - q11.2	5	0.453266	CHEK2P2, HERC2P3, GOLGA6L6, GOLGA8CP, NBEAP1, MIR3118-4, MIR3118-3, MIR3118-2, POTEB3, POTEB, POTEB2, NF1P2, MIR5701-3, MIR5701-1, MIR5701-2, LINC01193, LOC646214	Yes
20	Del	40,703,506	41,704,257	1,001	q12 - q13.11	20	-0.30984	PTPRT, LOC101927159	No
22	Del	47,716,755	49,920,184	2,203	q13.31 - q13.33	36	-0.27632	LL22NC03-75H12.2, LINC00898, LOC284930, MIR3201, FAM19A5, LOC284933, MIR4535, LINC01310, C22orf34	No

Total Amp/Del: 4

D

	All expressed	Primed TRIM28 enriched	Naive TRIM28 enriched
HERVH	1400	81	891
HERVK	622	169	287
SVA	2496	293	1225
LINE	139634	1055	5107

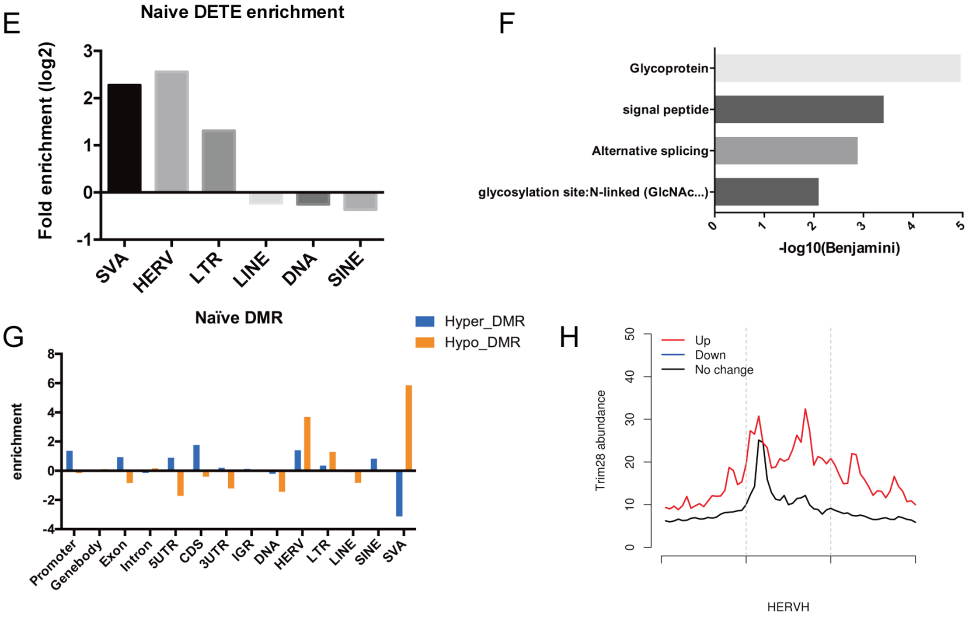

Figure S5 related to Figure 6

(A) Heatmap of diagnostic genes that were previously reported (Pastor et al., 2016) as being up-regulated in either the primed or naïve state as well as genes that are shared by both states.

(B) Average cell counts of over seven days of Ctrl (Ctrl UCLA1) and T28KO (T28KO(U1-9)) naïve hESCs (n=3 independent experiments), ** indicates p<0.01, error bar = SD.

(C) Copy Number Variants (CNV) analysis of naïve Ctrl and T28KO (U1-9) hESCs, naïve Ctrl hESCs have 2 CNVs which are overlapped with normal CNVs, naïve T28KO (U1-9) hESCs have another 2 deletions that are not overlapped with normal CNVs.

(D) Table shows numbers of all expressed HERVH, HERVK, SVA and LINE that are also bound by TRIM28 in control primed and control naïve hESCs using ChIP-Seq as defined by (Theunissen et al., 2016).

(E) Enrichment analysis of the DETE between Ctrl and T28KO (U1-9) naive hESC. Fold enrichment is represented as log2 (T28KO/Ctrl).

(F) Gene ontology analysis of up-regulated genes in T28KO (U1-9) naïve hESCs relative to Ctrls.

(G) Enrichment analysis of the differentially methylated regions (DMR) that have higher methylation (hyper_DMR) or lower methylation level (hypo_DMR) in T28KO (U1-9) naïve hESCs relative to Ctrl.

(H) TRIM28 binding of HERVH family members first defined as being up-regulated/down-regulated or unchanged in T28KO (U1-9) naïve hESCs relative to Ctrl. Notably, the up-regulated HERVH family members in T28KO naïve hESCs exhibit two peaks of TRIM28 binding, whereas the other groups do not.

Supplemental experimental procedures

Primed and naïve hESC culture

The primed hESC media was composed of 20% knockout serum replacement (KSR) (GIBCO), 100μM L-Glutamine (GIBCO), 1x MEM Non-Essential Amino Acids (NEAA) (GIBCO), 55μM 2-Mercaptoethanol (GIBCO), 10ng/mL recombinant human FGF basic (R&D systems), 1x Penicillin-Streptomycin (GIBCO), and 50ng/mL primocin (InvivoGen) in DMEM/F12 media (GIBCO). The primed hESCs were split by 1mg/ml Collagenase type IV (GIBCO) and maintained routinely on mitomycin C (MMC)-inactivated mouse embryonic fibroblasts (MEFs). Naïve hESCs were established by reverting the primed hESCs with 5iLAF media as described before (Theunissen et al., 2014). The composition of 5iLAF media was: DMEM/F-12 (Life Technologies) and Neurobasal (Life Technologies), with 1x N2 supplement (Life Technologies), 1x B27 supplement (Life Technologies), 8ng/mL bFGF (Peprotech), 1% nonessential amino acids (Life Technologies), 1mM GlutaMAX (Life Technologies), penicillin- streptomycin (Life Technologies), 0.1mM beta-mercaptoethanol (Life Technologies), 50μg/mL BSA (Sigma), 1μM PD0325901 (Stemgent), 1μM IM-12 (Enzo), 0.5μM SB590885 (R&D Systems), 1μM WH-4-023 (A Chemtek), 10μM Y-27632 (Stemgent), 20ng/mL Activin A (Peprotech), 20ng/mL rhLIF (Millipore) and 0.5% KSR (Life Technologies). The basic procedure was: primed cells were split to single cells by treatment with 0.05% Trypsin-EDTA (Life Technologies) for 3 minutes at 37ºC, after dissociation to single cells in hESC media+ 10uM ROCKi Y-27632 (Stemgent), cells were forced through a 40μm cell strainer, and 200K single cells were seeded onto MMC-treated feeders in hESC media+ROCKi; two days later, the media was changed to 5iLAF and replaced with fresh media every day until day 10, and the cells were then split again with Accutase (Innovative Cell Technologies), 200K cells were then seeded onto new feeders, colonies would start to form at subsequent passages, and naïve hESCs were then maintained in 5iLAF media.

Immunofluorescence staining

We performed immunofluorescence staining according to previous protocol (Gkountela et al., 2015; Li et al., 2015). Briefly cells were cultured on chamber slides, when reached 80%-90% confluency, cells were fixed with 4% paraformaldehyde in PBS for 10 minutes, washed with PBS containing 0.1% Tween 20 and permeabilized with PBS containing Triton-X100 for 10 min. Samples were blocked with 10% donkey serum for 30 minutes. Samples were incubated with goat-anti-OCT4 (Santa Cruze Biotechnology, sc-8628, 1:100), or Rabbit-anti-TRIM28 (Abcam, ab10483) overnight at 4ºC. The secondary antibodies were donkey-anti-rabbit-488 (Jackson ImmunoResearch Laboratories, 711-545-152) and donkey-anti-goat-594 (Jackson ImmunoResearch Laboratories, 705-586-147). DAPI was included in the mounting medium for indicating the nucleus.

Western blot

Cells were harvested and resuspended in 1x Laemmli buffer, and denatured for 10-15 minutes at 95ºC. Appropriate amount of samples were run on a NuPAGE 4-12% Bis-Tris gel (Invitrogen), gels were transferred at 200mA for 1.5 hours, and blots were blocked with 1xOdyssey blocking buffer overnight (Licor), followed by primary antibody incubation for 2 hours at room temperature, then secondary antibody incubation for 45 minutes at room temperature. The blots were then dried and imaged on Odyssey Infrared Imager (Licor). The primary antibodies using in this work are: rabbit-anti-TRIM28 (Abcam ab10483, 1:2000), mouse-anti-bACTIN (santa cruz sc47778, 1:1000), the secondary antibodies are 800CW-goat-anti-rabbit and 800CW-goat-anti-mouse (Licor, 1:20,000).

Naïve hESC flow cytometry analysis

Day 7 naïve hESCs were dissociated with 0.05% Trypsin-EDTA for 3-5 minutes at 37℃. The dissociated cells were stained with antibodies which were PE conjugated with TRA1-85 (R&D Systems) and SSEA4 conjugated with APC (R&D Systems) for at least 1hr on ice, then cells were washed with FACS buffer (1% BSA in PBS) once and resuspended in FACS buffer with DAPI (BD Pharmingen), finally cells were passed through 40μm cell strainer (Fisher scientific) and ready for flow cytometry analysis.

EB differentiation

Confluent hESCs were treated with Collagenase type IV (GIBCO) for 45-60 minutes at 37ºC, until the colonies start detaching from feeders when the plate was tapped, cells were collected and allowed to settle by gravity, then the supernant was carefully removed. The cells were resuspended with mTeSR (Stemcell Technologies) +10uM ROCKi (Stemgent) and then transfer to 6-well low attachment plate (Corning). 24 hours later media was changed to Differentiation Media which is composed of

DMEM/F12 media (Invitrogen/GIBCO), 20% ES FBS (Hyclone), 100µM L-Glutamine (GIBCO), 1x MEM Non-Essential Amino Acids (NEAA) (GIBCO), and 55µM 2-Mercaptoethanol (GIBCO), change media every 48 hours, collect cells at day 2 and day 5 for further analysis.

Real time quantitative PCR

Cell pellets were lysed in 350µL RLT buffer (QIAGEN) and RNA was extracted using RNeasy micro kit (QIAGEN, 74004). cDNA was synthesized using SuperScript® II Reverse Transcriptase (Invitrogen, 18064-014). Real time quantitative PCR was performed using TaqMan® Universal PCR Master Mix (Applied Biosystems, 4304437) and the expression levels of genes-of-interest were normalized to the expression of the housekeeping gene GAPDH. The Taqman probes are: GAPDH (Applied Biosystems, Hs99999905_m1), H19 (Applied Biosystems, Hs00399294_g1), MEG3 (Applied Biosystems, Hs00292028_m1).

ChIP-qPCR

ChIP assays were performed with approximately 1 million cells per experiment, based on a previously described protocol with slight modifications (Boyer et al., 2005). Briefly, cells were crosslinked with 1% formaldehyde for 10 mins at room temperature and formaldehyde was quenched by glycine to a final concentration of 0.125 M. Chromatin was sonicated to an average size of 0.5–2 kb by Bioruptor (Diagenode). A total of 3 mg of antibody was added to the sonicated chromatin and incubated overnight at 4ºC. 10% of chromatin used for each ChIP reaction was kept as an input DNA. Subsequently, 60µL of protein A magnetic Dynabeads (Invitrogen) were added to the ChIP reactions and incubated for two additional hours at 4ºC. Magnetic beads were washed and chromatin eluted, followed by reverse crosslinking and DNA purification. Resultant ChIP DNA was dissolved in elution buffer from Qiagen PCR Purification Kit.

For ChIP-qPCR, 2 sets of primers were designed for each locus, qPCR was performed as triple technical replicates, results were determined as percentage of input. Antibodies used for ChIP: TRIM28 (Abcam, ab10483), H3K9me3 (Abcam, ab8898). Primer sets for ChIP-qPCR: Primer set 1 (H19 locus): H19_1_Frw – CCATTTCAGGGTGTGGGGTG, H19_2_Rev – TTGCTGCTTGGACTAGGGTC. Primer set 3 (H19 locus): H19_3_Frw – GGTGTGCTGGGATGCTTTAAC, H19_4_Rev –CTTGACAATCAGCCTGGGTG. Primer set 11 (Meg3 promoter): MEG3_11_Frw – CCATACATGTGCAGGGTGAGAC, MEG3_12_Rev – GTCCTGGGTGTTCCTGAGGTATC. Primer set 13 (Meg3 promoter): MEG3_13_Frw– GACTAAGCCATACATGTGCAGG, MEG3_14_Rev – CCTGAGTGAGCTCTAGGCATTG.

Cardiomyocyte differentiation

Confluent hESCs were enzymatically detached and transferred into low attachment dish (Corning) with ESC Culture Medium as described earlier. Cells were cultured in serum-free cardiac differentiation medium which is composed of IMDM containing 1% MEM nonessential amino acid solution, 1% penicillinstreptomycin, 2 mM L-glutamine, 0.5 mM L-carnitine (Sigma-Aldrich), 0.001% 2-mercaptoethanol, and 0.4% human serum albumin (Sigma-Aldrich), with 4 mM CHIR (Axon) and 2 mM BIO (Calbiochem). On days 3–9, 10 mM KY02111 and/or other WNT inhibitors (XAV939 and/or IWP-2) were added to cell cultures, and the medium was changed every 2 days. On day 14, the cardiac colonies were collected for further analysis.

Bioinformatics analysis

RNA-seq analysis

mRNA reads were aligned to the human reference genome (hg19) using Tophat2 (Kim et al., 2013). The gene annotation was downloaded from iGenome (iGenome). The expression counts of genes were generated using HTSeq-count (Anders et al., 2015). To ensure the statistical power, genes have to be expressed (reads > 0) in at least 2 out of 3 replicates in both primed hESCs and naïve hESCs. To exclude the low expression genes, the most expressed replicate had to have a minimum of 10 reads. The annotation of TE was downloaded from (Theunissen et al., 2016) The expression counts of TEs were generated using multiBamCov from BEDtools (Quinlan and Hall, 2010). A TE was considered expressed, if it was expressed in one or more samples (RPKM>0). To exclude the low expression TEs, the most expressed replicate had to have a minimum of 10 reads (after normalizing for sequencing depth). Normalization for sequencing depth and differential gene/TE-expression analysis was performed using edgeR (Robinson et al., 2010). Genes or TEs with ≥2-fold difference in expression and FDR <5% were deemed differentially expressed.

Profiling genome-wide DNA methylation

Bisulfite converted reads were aligned to the human reference genome (hg19) using BS-Seeker 2 (Guo et al., 2013). Genome-wide DNA methylation profiles were generated by determining methylation levels for each cytosine in the genome. Since bisulfite treatment converts unmethylated cytosines (Cs) to thymines (Ts) after PCR amplification, the methylation level at each cytosine was determined as #C/(#C+#T), where #C represents the number of methylated reads and #T represents the number of unmethylated reads. The methylation level per cytosine serves as an estimate of the percentage of cells that have a methylated cytosine at a specific cytosine site. We only included cytosines that were covered by at least three reads. To identify differentially methylated regions (DMRs), a non-overlapping tiling window approach was used for genome-wide screening, where genomic regions of 500 bp containing ≥ 4 CpG sites in all compared samples were surveyed. A region is deemed differentially methylated if it shows ≥ 50% difference in average methylation level and a t-test p-value < 5%. A gene is considered differentially methylated (DMG) if it contains one or more DMRs in promoter or gene body.

ATAC-seq analysis

ATAC-seq reads were mapped to the human reference genome (hg19) using Bowtie2 (Langmead and Salzberg, 2012). Reads mapped to mitochondrial DNA were excluded from the analysis. For each sample the peaks of ATAC-seq reads are considered accessible regions, which were identified using MACS2 (Feng et al., 2012). To determine differential abundance of peaks between samples (i.e. regions that switch accessibility), the reads are normalized to 1X using deeptools (Ramírez et al., 2016). The resultant peak was considered differentially accessible if the foldchange of abundance at the peak between T28KO and Ctrl was ≥ 8, and a t-test p value < 5%.

ChIP-seq analysis

Raw reads of TRIM28 ChIP-seq downloaded from GSE84382 were mapped to the human reference genome (hg19) using Bowtie2 (Langmead and Salzberg, 2012). Peak calling was performed using MACS2 (Feng et al., 2012).

Supplemental References

Anders, S., Pyl, P.T., and Huber, W. (2015). HTSeq—a Python framework to work with high-throughput sequencing data. Bioinformatics *31*, 166-169.

Boyer, L.A., Lee, T.I., Cole, M.F., Johnstone, S.E., Levine, S.S., Zucker, J.P., Guenther, M.G., Kumar, R.M., Murray, H.L., Jenner, R.G.*, et al.* (2005). Core Transcriptional Regulatory Circuitry in Human Embryonic Stem Cells. Cell *122*, 947-956.

Feng, J., Liu, T., Qin, B., Zhang, Y., and Liu, X.S. (2012). Identifying ChIP-seq enrichment using MACS. Nat. Protocols *7*, 1728-1740.

Gkountela, S., Zhang, Kelvin X., Shafiq, Tiasha A., Liao, W.-W., Hargan-Calvopiña, J., Chen, P.-Y., and Clark, Amander T. (2015). DNA Demethylation Dynamics in the Human Prenatal Germline. Cell *161*, 1425-1436.

Guo, W., Fiziev, P., Yan, W., Cokus, S., Sun, X., Zhang, M.Q., Chen, P.-Y., and Pellegrini, M. (2013). BS-Seeker2: a versatile aligning pipeline for bisulfite sequencing data. BMC Genomics *14*, 774.

Kim, D., Pertea, G., Trapnell, C., Pimentel, H., Kelley, R., and Salzberg, S.L. (2013). TopHat2: accurate alignment of transcriptomes in the presence of insertions, deletions and gene fusions. Genome Biology *14*, R36.

Langmead, B., and Salzberg, S.L. (2012). Fast gapped-read alignment with Bowtie 2. Nat Meth *9*, 357-359.

Li, Z., Yu, J., Hosohama, L., Nee, K., Gkountela, S., Chaudhari, S., Cass, A.A., Xiao, X., and Clark, A.T. (2015). The Sm protein methyltransferase PRMT5 is not required for primordial germ cell specification in mice. The EMBO Journal *34*, 748-758.

Pastor, William A., Chen, D., Liu, W., Kim, R., Sahakyan, A., Lukianchikov, A., Plath, K., Jacobsen, Steven E., and Clark, Amander T. (2016). Naive Human Pluripotent Cells Feature a Methylation Landscape Devoid of Blastocyst or Germline Memory. Cell Stem Cell *18*, 323-329.

Quinlan, A.R., and Hall, I.M. (2010). BEDTools: a flexible suite of utilities for comparing genomic features. Bioinformatics *26*, 841-842.

Ramírez, F., Ryan, D.P., Grüning, B., Bhardwaj, V., Kilpert, F., Richter, A.S., Heyne, S., Dündar, F., and Manke, T. (2016). deepTools2: a next generation web server for deep-sequencing data analysis. Nucleic Acids Research *44*, W160-W165.

Robinson, M.D., McCarthy, D.J., and Smyth, G.K. (2010). edgeR: a Bioconductor package for differential expression analysis of digital gene expression data. Bioinformatics *26*, 139-140.

Theunissen, Thorold W., Friedli, M., He, Y., Planet, E., O'Neil, Ryan C., Markoulaki, S., Pontis, J., Wang, H., Iouranova, A., Imbeault, M.*, et al.* (2016). Molecular Criteria for Defining the Naive Human Pluripotent State. Cell Stem Cell.
